# Assessment of Efficacy and Tolerability of Medicinal Cannabinoids in Patients With Multiple Sclerosis: A Systematic Review and Meta-analysis

**DOI:** 10.1001/jamanetworkopen.2018.3485

**Published:** 2018-10-12

**Authors:** Mari Carmen Torres-Moreno, Esther Papaseit, Marta Torrens, Magí Farré

**Affiliations:** 1 Universitat Autònoma de Barcelona, Departament de Farmacologia, Terapèutica i Toxicologia, Cerdanyola del Vallès, Spain; 2 Hospital Universitari Germans Trias i Pujol and Institut de Recerca Germans Trias i Pujol, Servei de Farmacologia Clínica, Badalona, Spain; 3 Universitat Autònoma de Barcelona, Departament de Psiquiatria i Medicina Legal, Cerdanyola del Vallès, Spain; 4 Hospital del Mar, Institut de Neuropsiquiatria i Addiccions, Programa Addiccions, Barcelona, Spain

## Abstract

**Importance:**

Cannabinoids have antispastic and analgesic effects; however, their role in the treatment of multiple sclerosis (MS) symptoms is not well defined.

**Objective:**

To conduct a systematic review and meta-analysis to assess the efficacy and tolerability of medicinal cannabinoids compared with placebo in the symptomatic treatment of patients with MS.

**Data Sources:**

MEDLINE and the Cochrane Library Plus up to July 26, 2016. No restrictions were applied. The search was completed with information from ClinicalTrials.gov.

**Study Selection:**

Randomized, double-blind, and placebo-controlled trials evaluating the effect of medicinal cannabinoids by oral or oromucosal route of administration on the symptoms of spasticity, pain, or bladder dysfunction in adult patients with MS.

**Data Extraction and Synthesis:**

The Preferred Reporting Items for Systematic Reviews and Meta-analyses (PRISMA) reporting guidelines were followed. Effect sizes were calculated as standardized mean difference (SMD) for efficacy, and rate ratio (RR) for tolerability. Within each study, those SMDs evaluating the same outcome were combined before the meta-analysis to obtain a single value per outcome and study. Pooling of the studies was performed on an intention-to-treat basis by means of random-effect meta-analysis.

**Main Outcomes and Measures:**

Spasticity (on the Ashworth and Modified Ashworth scales and subjective), pain, bladder dysfunction, adverse events, and withdrawals due to adverse events.

**Results:**

Seventeen selected trials including 3161 patients were analyzed. Significant findings for the efficacy of cannabinoids vs placebo were SMD = −0.25 SD (95% CI, −0.38 to −0.13 SD) for spasticity (subjective patient assessment data), −0.17 SD (95% CI, −0.31 to −0.03 SD) for pain, and −0.11 SD (95% CI, −0.22 to −0.0008 SD) for bladder dysfunction. Results favored cannabinoids. Findings for tolerability were RR = 1.72 patient-years (95% CI, 1.46-2.02 patient-years) in the total adverse events analysis and 2.95 patient-years (95% CI, 2.14-4.07 patient-years) in withdrawals due to adverse events. Results described a higher risk for cannabinoids. The serious adverse events meta-analysis showed no statistical significance.

**Conclusions and Relevance:**

The results suggest a limited efficacy of cannabinoids for the treatment of spasticity, pain, and bladder dysfunction in patients with MS. Therapy using these drugs can be considered as safe.

**Trial Registration:**

PROSPERO Identifier: CRD42014015391

## Key Points

**Question:**

Are medicinal cannabinoids effective and well tolerated in the treatment of multiple sclerosis?

**Findings:**

In this systematic review and meta-analysis of 17 randomized clinical trials including 3161 patients, cannabinoids were significantly associated with efficacy for subjective spasticity, pain, and bladder dysfunction compared with placebo. Cannabinoids had a higher risk of adverse events and withdrawals due to adverse events, with no statistically significant differences found for serious adverse events.

**Meaning:**

Cannabinoids appear to be safe regarding serious adverse events, but their clinical benefit may be limited.

## Teaser

This systematic review and meta-analysis of 17 randomized clinical trials including 3161 patients examines the association of efficacy and tolerability of cannabinoids with spasticity, pain, and bladder dysfunction in patients with multiple sclerosis (MS).

## Introduction

Multiple sclerosis (MS) is a neurodegenerative disease characterized by demyelination in the central nervous system caused by inflammatory immune-mediated attacks. In 2013 there were approximately 2.3 million people affected by MS worldwide.^1^ Manifestations may occur in an episodic (relapsing-remitting) or progressive (primary or secondary) pattern and vary from benign to severe. Sensory and motor systems are frequently affected and present symptoms of spasticity, pain, and bladder dysfunction.^2^ Treatment of MS focuses on preventing new relapses, modifying the course of the disease, and managing symptoms. No treatment to stimulate remyelination and repair nerves is available.^3^

Cannabinoids act as neuromodulators of the endocannabinoid system; therefore, their therapeutic potential has aroused considerable interest over the centuries. In some countries a mixture of cannabinoids (nabiximols) has been approved for the symptomatic treatment of MS spasticity and neuropathic pain in cases in which previous medication has proved ineffective.^4,5^ Nabiximols are a mixture of δ-9-tetrahydrocannabinol (THC) and cannabidiol (CBD) in an approximate ratio of 1:1. Oral cannabis extract (CE) contains THC and CBD from the *Cannabis sativa* plant. Other marketed cannabinoids include dronabinol, an oral synthetic THC, and nabilone, an oral synthetic THC analogue.

Limited literature regarding previous systematic reviews and meta-analysis was found.^6,7,8,9,10,11^ The results from these studies were relatively incomplete.

We aimed to evaluate the therapeutic efficacy and tolerability of medicinal cannabinoids to treat the symptoms of spasticity, pain, and bladder dysfunction in patients with MS by performing a systematic review and meta-analysis of randomized, double-blind, and placebo-controlled trials.

## Methods

### Study Protocol

A protocol of the study was prepared and recorded in the International Prospective Register of Systematic Reviews (PROSPERO). The review was undertaken according to the Preferred Reporting Items for Systematic Reviews and Meta-analyses (PRISMA) reporting guidelines.^12^

### Study Eligibility Criteria

The inclusion criteria were (1) published studies evaluating the effect of medicinal cannabinoids by oral or oromucosal route on the symptoms of spasticity, pain, or bladder dysfunction in adult patients with MS; (2) randomized, placebo-controlled, double-blind, and parallel or crossover designed trials; (3) a minimum length of treatment of 2 weeks; and (4) studies specifying the results by means of estimated effect size or with sufficient information to calculate it. The exclusion criteria were (1) studies investigating other clinical entities and (2) studies duplicated in publication.

### Search and Selection of Studies

Search, study selection, and data collection were jointly conducted by 2 of us (M.C.T.M. and M.F). The summary of the studies was read by these 2 authors; in case of disagreement, the study was again reviewed, and a final decision was reached by consensus.

The bibliographic search was carried out up to July 26, 2016, in the electronic databases MEDLINE and the Cochrane Library Plus. No limits regarding publication date, article type, or language were applied. An additional search was performed in ClinicalTrials.gov to obtain complementary information not provided in the articles. The search strategy used was “(canna* OR tetrahydrocannabinol OR THC OR marijuana OR dronabinol OR nabilone OR levonantradol OR dexanabinol OR sativex OR namisol OR marihuana OR cesamet OR marinol OR nabiximols) AND multiple sclerosis.” The abstracts were reviewed to identify randomized clinical trials (RCTs). The references of the reviews and selected studies were checked to identify other RCTs that had not been located. In parallel, other documents such as books, monographs, and reports were also reviewed. The authors of the identified studies were contacted in the case of controversy to clarify appropriateness for inclusion.

### Data Extraction

All available data were collected to select those that were valid to compare efficacy and tolerability from the published articles found in the electronic databases and complemented with results from ClinicalTrials.gov. The general data selection criteria were (1) information measuring efficacy and tolerability and (2) information about the phases fulfilling the inclusion criteria in the case of studies with different phases.

The data selection criteria for efficacy were (1) data convertible to the effect size of standardized mean difference (SMD) and (2) data from the tools measuring the same clinical aspects. For tolerability, the selection criteria were (1) the number of adverse events or, in case of failure, the number of patients presenting an adverse event, appearing in at least 2 of the studies and (2) the number of patients withdrawn from the intervention and/or the study due to adverse events.

### Assessment of Bias of Studies

Estimates of the risk of bias of each of the included studies, and across them, were reached according to the recommendations of the Cochrane Collaboration. Ratings were low risk of bias, high risk of bias, and unclear risk of bias. Each study was reviewed individually. Assessment of publication bias for each meta-analysis was also performed. Assessments were carried out using the software Review Manager (RevMan) (Cochrane).^13^

### Synthesis

In efficacy, high heterogeneity was clearly demonstrated in the format by which results were obtained (eg, *F *statistic, mean difference between groups, or odds ratio), making a direct comparison nonviable. As a consequence, standardization to the SMD, which is expressed in standard deviation units, was calculated in order to allow comparison. The SMD used was Hedges *g* and hereafter the SMD referred to in our study is this unless otherwise indicated. The related standard error was also estimated. Effect size can be interpreted in the clinical field following the rule of thumb in which values of 0.2, 0.5, and 0.8 represent small, moderate, and large effect, respectively.^14^ Calculations of the SMD were carried out on an intention-to-treat (ITT) basis by extrapolation of the missing data.^14,15,16,17,18,19,20,21,22^ Crossover studies were treated as parallel design.^23^ With respect to the evaluation of efficacy, it was necessary to modify the direction of some clinical tools to adapt the results, as some were using higher or inverse punctuations for clinical improvement.

The primary studies provided multiple data results obtained from different clinical assessment tools (eg, pain measurement with a numerical rating scale, visual analog scale, and the Neuropathic Pain Scale) for the same common outcome. Converted data resulting from these tools within the same study were combined to include as many data as possible and to avoid loss of information. This option also reduces the risk of bias due to the subjective selection of 1 unique clinical measure on our part. After combination, a single SMD value per outcome and study was obtained, ensuring the assumption of independence of effect sizes. Data pooling was carried out by the simple averages of the SMDs and their standard errors.^18,24^

For tolerability, data were analyzed in the form of the rate ratio (RR).^14^

The meta-analysis was performed with RevMan software using the inverse-of-variance method. The random-effects model was used on an ITT basis. For efficacy, SMDs and their standard errors were analyzed. For tolerability outcomes, the natural logarithm (ln) of the RRs and its respective standard errors were introduced. The heterogeneity of the results was evaluated by means of the *I*^2^ statistic.

### Sensitivity Analysis

After the systematic review, we conducted a sensitivity analysis of the results obtained to ascertain whether the findings were strong enough to reaffirm the methods used. With this objective, the meta-analyses were repeated, changing the parameters that could be affected by our decisions: (1) use of the fixed-effects model instead of random effects; (2) exclusion of crossover studies; (3) exclusion of studies with a sample size of 50 patients or fewer; (4) exclusion of studies with a length of treatment of 4 weeks or less; and (5) exclusion of studies with a high risk of bias in any of the evaluated domains. Furthermore, to reaffirm our calculations, other parallel secondary estimations for SMDs were performed with data from the studies.

## Results

### Study Characteristics

A total of 17 RCTs (19 articles) from 775 records were included in our study.^25,26,27,28,29,30,31,32,33,34,35,36,37,38,39,40,41,42,43^ Another 22 of the 775 records underwent full-text review but were later excluded (eReferences in the Supplement). Regarding the 17 RCTs, 2 had 2 articles each. In the statistical analysis, they are referred to as Zajicek,^26^ 2003/Freeman,^27^ 2006 and Aragona,^33^ 2009/Tomassini,^34^ 2014. Two of the studies were conducted in 2 phases (A and B).^37,39^ Only phase B was analyzed in 1 of the studies, in which participants were responders after the initial phase A treatment.^37^ The initial participants in phase A of the other study were included, in that case excluding phase B.^39^ One of the selected articles^42^ was based on a published RCT (reference e2 in eReferences in the Supplement) that was discarded after reading the full text, owing to lack of data results. Of the 17 RCTs, 5 (6 articles) were crossover design.^25,28,29,33,34,43^ The total number of patients analyzed was 3161. The studied experimental interventions were (1) oral CE^25,26,27,29,38^; (2) oromucosal CE (nabiximols)^30,31,32,33,34,35,36,37,39,40,43^; (3) oral dronabinol^25,26,27,28,42^; and (4) oral nabilone,^41^ evaluated as an adjunctive treatment to gabapentin. Two of the studies (3 articles) included 2 experimental groups, using both oral CE and dronabinol in comparison with placebo. Each experimental-placebo comparison was included separately.^25,26,27^

**Figure 1.**
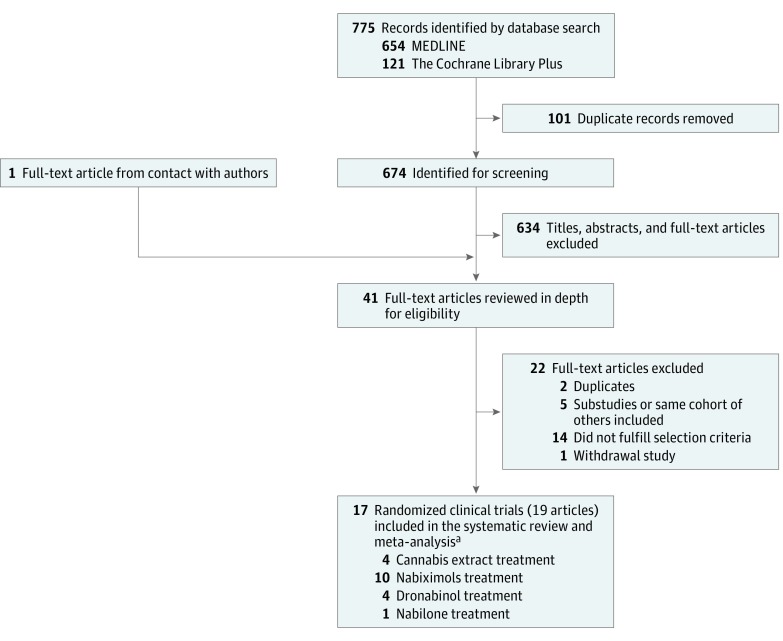
Study Selection Flowchart ^a^Two studies using both cannabis extract and dronabinol as experimental treatments.

Figure 1 shows selection of included studies. Main characteristics and outcome measures of each study are included in Table 1; eTable 1 in the Supplement shows further detail. Hereafter, all the results of the pooled-effect sizes of the previously mentioned treatments within the respective meta-analyses are referred to as cannabinoids, unless otherwise indicated.

**Table 1.** Summary of Characteristics of the Included Studies

Source	Design^a^	Patients, No.	Interventions, Mean (SD) Dose		
			THC/CBD (CE or Nabiximols)	THC (Dronabinol or Nabilone)	Placebo
Killestein et al,^25^ 2002	Patients with progressive MS with spasticity, setting not specified, crossover, 20 wk (4-wk intervention, 4-wk washout between treatment periods), ITT analysis	16	CE: dose = 2-4 caps/d (5-10 mg THC + approximately 1.25-2.50 mg CBD)	Dronabinol: Dose = 2-4 caps/d (5-10 mg THC)	Dose = 2-4 caps/d
Zajicek et al,^26^ 2003 (same study cohort as Freeman et al,^27^ 2006)	Patients with MS with spasticity, multicentric (UK), parallel, 15 wk, ITT analysis	630	CE: 5.42 (2.11) caps/d (13.56 mg THC + 6.78 mg CBD)^b^	Dronabinol: 5.47 (2.08) caps/d (13.67 mg THC)^b^	6.24 (1.71) caps/d^b^
Freeman et al,^27^ 2006 (same study cohort as Zajicek et al,^26^ 2003)	Patients recruited to the Zajicek et al,^26^ 2003 study, except those with a permanent catheter	522 (83% of Zajicek et al,^26^ 2003 initial data)	CE: 5.42 (2.11) caps/d (13.56 mg THC + 6.78 mg CBD)^b^	Dronabinol: 5.47 (2.08) caps/d (13.67 mg THC)^b^	6.24 (1.71) caps/d^b^
Svendsen et al,^28^ 2004	Patients with MS with central neuropathic pain, unicentric (Denmark), crossover, 9 wk (3-wk intervention, 3-wk washout between treatment periods), ITT analysis	24	NA	Dronabinol: Mean (range) dose, 3.1 (2.7-3.6) caps/d (7.75 [6.75-9.00] mg THC)	Mean (range) dose, 3.3 (2.8-3.6) caps/d (8.25 [7.00-22.50] mg)
Vaney et al,^29^ 2004	Patients with MS with spasticity, unicentric (Switzerland), crossover, 4 wk (2-wk CE treatment, 1-wk placebo, 3-d washout between and after interventions), ITT and PP analyses	57	CE: 7.20 caps/d (17.99 [7.63] mg THC + 6.48 [2.75] mg CBD)^b^	NA	Mean dose not specified
Wade et al,^30^ 2004	Patients with MS with spasticity, spasms, bladder problems, tremor, or pain (not musculoskeletal); multicentric (UK); parallel; 6 wk; PP analysis	160	Nabiximols: 12.37 (6.05) sprays/d (33.40 mg THC + 30.93 mg CBD)^b^	NA	18.87 (6.17) sprays/d^b^
Rog et al,^31^ 2005	Patients with MS with central neuropathic pain, unicentric (UK), parallel, 5 wk, ITT analysis	66	Nabiximols: 9.6 (6.1) sprays/d (wk 4) (25.92 mg THC + 24.00 mg CBD)	NA	19.1 (12.9) sprays/d (wk 4)
Collin et al,^32^ 2007	Patients with MS with spasticity, multicentric (UK and Romania), parallel, 6 wk, ITT and PP analyses	189	Nabiximols: 9.4 (6.4) sprays/d (25.38 mg THC + 23.50 mg CBD)	NA	14.7 (8.4) sprays/d
Aragona et al,^33^ 2009 (same study cohort as Tomassini et al,^34^ 2014)	Patients with secondary progressive MS with spasticity, unicentric (Italy), crossover, 10 wk (3-wk intervention, 2-wk washout between and after treatment periods), ITT and PP analyses	17 (94% with respect to Tomassini et at,^34^ 2014 initial data)	Nabiximols: 8.20 (3.15) sprays/d (22.14 mg THC + 20.50 mg CBD)	NA	15.16 (4.51) sprays/d
Tomassini et at,^34^ 2014 (same study cohort as Aragona et al,^33^ 2009)	Patients with secondary progressive MS with spasticity, unicentric (Italy), crossover, 10 wk (3-wk intervention, 2-wk washout between and after treatment periods), ITT and PP analyses	18	Nabiximols: median (range) dose, 7.4 (2.7-12.5) sprays/d (19.98 mg THC + 18.50 mg CBD)	NA	Median (range) dose, 16.1 (6.7-26.0) sprays/d
Collin et al,^35^ 2010	Patients with MS with spasticity, multicentric (UK and Czech Republic), parallel, 15 wk, ITT and PP analyses	337	Nabiximols: mean (range) dose, 8.5 (1-22) sprays/d (22.95 mg THC + 21.25 mg CBD)	NA	Mean (range) dose, 15.4 (2-23) sprays/d
Kavia et al,^36^ 2010	Patients with MS with overactive bladder, multicentric (UK, Belgium, and Romania), parallel, 10 wk, ITT and PP analyses	135	Nabiximols: mean (median) dose, 8.91 (7.19) sprays/d (24.06 mg THC + 22.28 mg CBD)	NA	Mean (median) dose,17.05 (14.22) sprays/d
Novotna et al,^37^ 2011 (phase B)	Patients with MS with spasticity and at least a 20% reduction in mean spasticity numerical rating scale score after the previous single-blind phase A treatment (responders), multicentric (UK, Spain, Poland, Czech Republic, and Italy), parallel, 12 wk, ITT and PP analyses	241	Nabiximols: 8.3 (2.43) sprays/d (22.41 mg THC + 20.75 mg CBD)	NA	8.9 (2.31) sprays/d
Zajicek et al,^38^ 2012	Patients with MS with muscle stiffness, multicentric (UK), parallel, 14 wk, ITT analysis	277	CE: 7.81 (2.75) caps/d (end of titration period) (19.52 mg THC + 9.76 mg CBD); 6.81 (2.99) caps/d (end of study period) (17.02 mg THC + 8.51 mg CBD)^b^	NA	9.60 (1.27) caps/d (end of titration period) (24.00 mg); 9.36 (1.51) caps/d (end of study period) (23.40 mg)^b^
Langford et al,^39^ 2013 (phase A)	Patients with MS with central neuropathic pain, multicentric (UK, Czech Republic, Canada, Spain and France), parallel, 15 wk, ITT and PP analyses	339	Nabiximols: 8.8 (3.87) sprays/d (23.76 mg THC + 22.00 mg CBD)	NA	11.1 (4.6) sprays/d
Vachová et al,^40^ 2014	Patients with MS with spasticity, multicentric (Czech Republic), parallel, 50 wk, ITT and PP analyses	121	Nabiximols: 7.6 (3.1) sprays/d (first mo) (20.52 mg THC + 19.00 mg CBD); 6.4 (3.1) sprays/d (last 3 mo) (17.28 mg THC + 16.00 mg CBD)	NA	9.5 (2.4/2.6) sprays/d (from first to last 3 mo)
Turcotte et al,^41^ 2015	Patients with relapsing-remitting MS and neuropathic pain receiving a stable dose of gabapentin (≥1800 mg/d), unicentric (Canada), parallel, 9 wk, ITT and PP analyses	15	NA	Nabilone: dose = 1-2 caps/d (0.5-1 mg THC/caps) (0.5-2 mg THC)	Dose = 1-2 caps/d (0.5-1 mg/caps) (0.5-2 mg)
Ball et al,^42^ 2015	Patients with progressive MS, multicentric (UK), parallel, 3 y, ITT and PP analyses	493	NA	Dronabinol: median (IQR) dose, 4 (2-6) caps/d (final y of follow-up) 14.00 mg THC	Median (IQR) dose, 6 (4-8) caps/d (final y of follow-up)
Leocani, et al,^43^ 2015	Patients with progressive MS with lower limb spasticity, unicentric (Italy), crossover, 10 wk (4-wk intervention, 2-wk washout between treatment periods), PP analysis	43	Nabiximols: 7 (3) sprays/d (18.90 mg THC + 17.50 mg CBD)	NA	10 (3) sprays/d

Abbreviations: CBD, cannabidiol; CE, *Cannabis sativa* plant extract; IQR, interquartile range; ITT, intention-to-treat; MS, multiple sclerosis; NA, not applicable; PP, per-protocol; THC, δ-9-tetrahydrocannabinol; UK, United Kingdom.

^a^ Randomized, placebo-controlled, double-blind study.

^b^ Estimated by the authors of this systematic review from study data.

### Bias of Studies

The risk-of-bias summary of each study included in the systematic review is depicted in eFigure 1 in the Supplement. According to the authors’ judgement, high risk of bias was found relative to blinding of participants and personnel,^37^ blinding of outcome assessment,^33,34,41,43^ incomplete outcome data,^26,27,41,42^ and selective reporting,^25^ with the greatest percentage of high risk for bias concerning blinding of outcome assessment and incomplete outcome data (eFigure 2 in the Supplement). The impact on our results was evaluated in the sensitivity analysis. Publication bias analyses for each meta-analysis are shown in eFigure 3 in the Supplement for efficacy outcomes and eFigure 4 in the Supplement for tolerability. Publication bias was detected both for and against cannabinoids.

### Efficacy

A total of 82 results from clinical assessment tools were selected and converted to SMDs, and 17 combinations were carried out among them. A summary of all selected clinical assessment tools can be seen in eTable 2 in the Supplement. Clinical effect in favor of the experimental treatment is denoted by a negative SMD, and a positive value favors the placebo. Statistically significant results are considered favorable for cannabinoids or placebo whenever the confidence interval of the results does not exceed the value of no effect (0 in case of the SMD).

**Figure 2.**
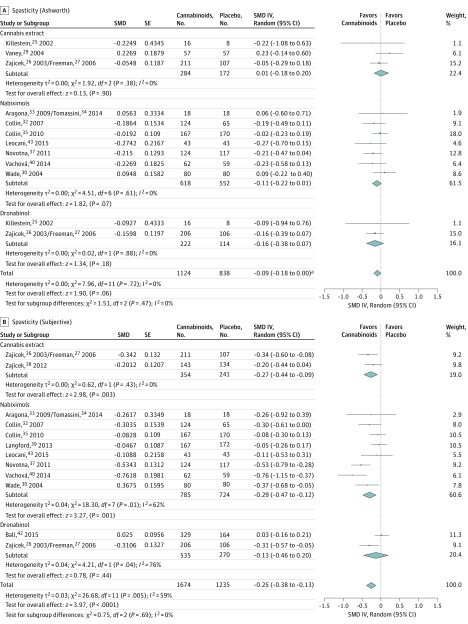
Analysis of Efficacy The central point of the bars and diamonds indicates the magnitude of the effect size (Hedges *g* standardized mean difference [SMD] value), while width indicates the 95% CI. IV indicates inverse of variance. ^a^Upper confidence interval value of 0.0027.

Spasticity was evaluated separately for objective measures scored by an observer on the Ashworth and Modified Ashworth scales (referred to as *spasticity [Ashworth]*), and for the subjective spasticity measures (patient assessment data). No effects of cannabinoids on the Ashworth and Modified Ashworth scales were observed. Results showed statistically significant differences in favor of the experimental group vs placebo in spasticity (subjective) in CE (SMD, −0.27 SD; 95% CI, −0.44 to −0.09 SD), nabiximols (SMD, −0.29 SD; 95% CI, −0.47 to −0.12 SD), and cannabinoids (SMD, −0.25 SD; 95% CI, −0.38 to −0.13 SD). Figure 2A shows the meta-analysis for spasticity (Ashworth), and Figure 2B for spasticity (subjective).

**Figure 3.**
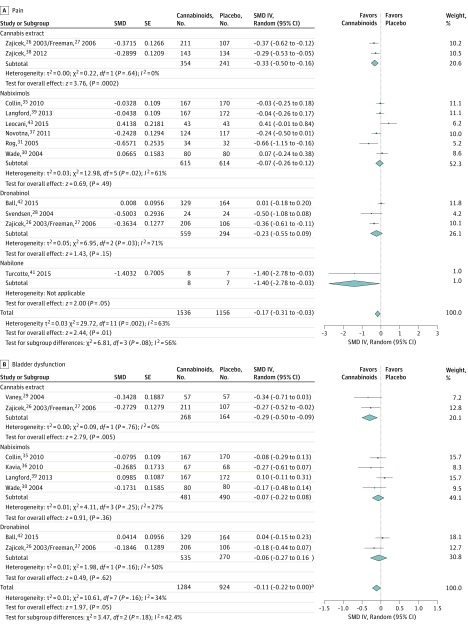
Analysis of Efficacy The central point of the bars and diamonds indicates the magnitude of the effect size (Hedges *g* standardized mean difference [SMD] value), while width indicates the 95% confidence interval. IV indicates inverse of variance. ^a^Upper confidence interval value of −0.0008.

Results in pain presented statistically significant differences in favor of CE (SMD, −0.33 SD; 95% CI, −0.50 to −0.16 SD), nabilone (SMD, −1.40 SD; 95% CI, −2.78 to −0.03 SD), and cannabinoids (SMD, −0.17 SD; 95% CI, −0.31 to −0.03 SD). Figure 3A shows the meta-analysis for pain. Similar results were obtained for bladder dysfunction in CE (SMD, −0.29 SD; 95% CI, −0.50 to −0.09 SD) and cannabinoids (SMD, −0.11 SD; 95% CI, −0.22 to −0.0008 SD) (Figure 3B).

One possible concern in clinical trial results is the impact of industry-funded studies. In our meta-analysis, all the studies concerning CE^25,26,27,29,38^ and dronabinol^28,42^ were funded by independent grants. The study of nabilone^41^ and all of those concerning nabiximols^30,31,32,35,36,37,39,40,43^ (except 1^33,34^) were funded by pharmaceutical companies (nabilone by Valeant Canada and nabiximols by GW Pharma and Laboratorios Almirall). We performed an additional analysis excluding those industry-funded studies. The additional analysis showed no differences between nabiximols and placebo in all the efficacy outcomes. For spasticity (Ashworth), the values changed from −0.11 SD (95% CI, −0.22 to 0.01 SD) to 0.06 SD (95% CI, −0.60 to 0.71 SD); for subjective spasticity, the values changed from −0.29 SD (95% CI, −0.47 to −0.12 SD) to −0.26 SD (95% CI, −0.92 to 0.39 SD); and for pain and bladder dysfunction, the values changed to not estimable. The same occurred in nabilone, for which the effect on pain could not be estimated. In the analysis for cannabinoids, only results for bladder dysfunction changed in terms of statistical significance, becoming nonsignificant. It seems that sponsored studies favored active treatment.

### Tolerability

A total of 5357 adverse events were selected to be analyzed. Serious adverse events (death or threat to a patient's life or functioning) were also calculated, with 325 events included. A total of 260 withdrawals were due to adverse events. Higher risk in the experimental treatment is denoted by an RR greater than 1, while an RR less than 1 is for placebo. Results are considered statistically significant with higher risk in cannabinoids or placebo whenever the confidence interval of the results does not exceed the value of no effect (1 in the case of the RR).

In the total adverse events analysis, there was a higher risk of adverse events in active treatments vs placebo in nabiximols (RR, 1.80 patient-years; 95% CI, 1.53-2.12 patient-years), dronabinol (RR, 1.62 patient-years; 95% CI, 1.12-2.34 patient-years), and cannabinoids (RR, 1.72 patient-years; 95% CI, 1.46-2.02 patient-years) and a higher risk of withdrawals due to adverse events in CE (RR, 3.11 patient-years; 95% CI, 1.54-6.28 patient-years), nabiximols (RR, 2.20 patient-years; 95% CI, 1.34-3.59 patient-years), dronabinol (RR, 4.12 patient-years; 95% CI, 2.39-7.11 patient-years), and cannabinoids (RR, 2.95 patient-years; 95% CI, 2.14-4.07 patient-years), but not in nabilone. No statistical significance was found in the meta-analysis of serious adverse events. Additionally, results showed a higher risk in cannabinoids with respect to the adverse events of dizziness or vertigo, dry mouth, fatigue, feeling drunk, impaired balance or ataxia, memory impairment, and somnolence. Table 2 shows the results obtained after analysis of the tolerability data.

**Table 2.** Tolerability Results

Outcome	Interventions															
	Cannabis Extract			Nabiximols			Dronabinol			Nabilone			Cannabinoids			Statistically Significant Higher Risk
	RR (95% CI)	Studies, No.	Patients, No.	RR (95% CI)	Studies, No.	Patients, No.	RR (95% CI)	Studies, No.	Patients, No.	RR (95% CI)	Studies, No.	Patients, No.	RR (95% CI)	Studies, No.	Patients, No.	
Total adverse events	1.51 (0.87-2.63)	4^25,26,27,29,38^	733	1.80 (1.53-2.12)	10^30,31,32,33,34,35,36,37,39,40,43^	1710	1.62 (1.12-2.34)	4^25,26,27,28,42^	877	NA	NA	NA	1.72 (1.46-2.02)	16	3320	Nabiximols, dronabinol, cannabinoids
Serious adverse events	0.99 (0.26-3.74)	2^26,27,38^	595	1.43 (0.66-3.09)	8^30,32,35,36,37,39,40,43^	1608	1.21 (0.89-1.63)	3^26,27,28,42^	853	NA	NA ·	NA	1.23 (0.82-1.85)	12	3056	NS
Withdrawals due to adverse events	3.11 (1.54-6.28)	3^26,27,29,38^	709	2.20 (1.34-3.59)	9^30,31,32,35,36,37,39,40,43^	1674	4.12 (2.39-7.11)	2^26,27,42^	805	2.63 (0.11-64.44)	1^41^	15	2.95 (2.14-4.07)	14	3203	Cannabis extract, nabiximols, dronabinol, cannabinoids
Dizziness or vertigo	2.51 (0.84-7.47)	4^25,26,27,29,38^	733	3.33 (2.55-4.34)	10^30,31,32,33,34,35,36,37,39,40,43^	1710	4.00 (2.43-6.58)	4^25,26,27,28,42^	877	NA	NA	NA	3.40 (2.55-4.53)	16	3320	Nabiximols, dronabinol, cannabinoids
Dry mouth	3.17 (1.91-5.25)	4^25,26,27,29,38^	733	2.30 (1.42-3.73)	8^30,31,32,33,34,35,37,39,40^	1489	4.32 (2.12-8.81)	3^25,26,27,28^	384	NA	NA·	NA	2.94 (2.15-4.03)	13	2606	Cannabis extract, nabiximols, dronabinol, cannabinoids
Fatigue	2.60 (1.22-5.58)	1^38^	277	1.64 (1.17-2.28)	9^30,31,32,33,34,35,36,37,39,40^	1624	1.09 (0.74-1.60)	2^28,42^	541	NA	NA	NA	1.61 (1.18-2.21)	12	2442	Cannabis extract, nabiximols, cannabinoids
Feeling drunk	NA	NA	NA	3.70 (0.70-19.55)	3^30,31,36^	361	11.00 (0.61-198.93)	1^28^	48	NA	NA	NA	4.85 (1.15-20.53)	4	409	Cannabinoids
Impaired balance or ataxia	3.50 (0.18-67.77)	1^25^	24	2.93 (1.04-8.27)	5^32,36,37,39,40^	1025	1.28 (0.90-1.81)	2^28,42^	541	NA	NA	NA	1.40 (1.01-1.95)	8	1590	Nabiximols, cannabinoids
Memory impairment	NA	NA	NA	4.93 (1.07-22.70)	3^36,39,40^	595	NA	NA	NA	NA	NA	NA	4.93 (1.07-22.70)	3	595	Nabiximols, cannabinoids
Somnolence	1.32 (0.95-1.83)	3^25,26,27,29^	456	3.47 (2.10-5.73)	10^30,31,32,33,34,35,36,37,39,40,43^	1710	0.55 (0.06-4.74)	2^25,26,27^	336	NA	NA	NA	1.87 (1.24-2.81)	13	2502	Nabiximols, cannabinoids

Abbreviations: NA, not available; NS, nonsignificant; RR, rate ratio.

### Sensitivity Analysis

In efficacy, 11.3% of the results in the sensitivity analysis (considering all estimated effect sizes of the interventions [CE, nabiximols, dronabinol, nabilone, and cannabinoids] and the 5 sensitivity analyses globally) became statistically significant or not, with respect to the main results. In tolerability, this percentage was 8.4%. A summary of the main and sensitivity analysis results is shown in eTables 3 and 4 in the Supplement, for efficacy and tolerability, respectively. Additionally, in efficacy, the mean (SE) of the overall differences between the main and secondary calculations to SMDs was −0.0019 (0.0014) SD.

## Discussion

To our knowledge, this is the most complete systematic review and meta-analysis of the effect of cannabinoids on MS. Our results show limited therapeutic efficacy of cannabinoids for the primary outcomes of spasticity, pain, and bladder dysfunction in patients with MS.

None of the interventions demonstrated clear efficacy in the treatment of spasticity when evaluated in a more objective form (ie, the Ashworth and Modified Ashworth scales). In the analysis of subjective spasticity, significant differences were observed with respect to the active treatments of CE, nabiximols, and cannabinoids. However, a large allocation-dependent placebo effect can be expected because of possible difficulties in masking and blinding. It is also interesting to note that the single largest (almost 500 patients), longest (up to 3 years), and non–corporate-sponsored study^42^ favored placebo with respect to its tested outcomes (spasticity [subjective], pain, and bladder dysfunction). Differences among results might stem from the fact that a minor improvement in such a disabling symptom is reflected by a more positive evaluation from the patient. Efficacy in pain of CE, nabilone, and cannabinoids was also demonstrated, in addition to efficacy in bladder dysfunction for CE and cannabinoids. Most of the therapeutic effects show a small value of SMD, approximately between −0.09 and −0.25 SD, which represents a limited (small) therapeutic effect.^14^

Six previous systematic reviews performed meta-analyses to evaluate the efficacy of cannabinoids in MS symptoms.^6,7,8,9,10,11^ One study evaluated spasticity (Wade et al^6^) and another, pain (Iskedjian et al^7^) outcomes; 3 analyzed both spasticity and pain (Whiting et al,^8^ Meza et al,^9^ da Rovare et al^10^). One of these studies (da Rovare et al^10^) and Abo Youssef et al^11^ evaluated bladder dysfunction. Three of them did not focus only on patients with MS in the spasticity and pain analyses (Iskedjian et al,^7^ Whiting et al,^8^ da Rovare et al^10^).

Our results are in accordance with the first 3 systematic reviews^6,7,8^ and with the last one,^11^ taking into account differences in treatments and analyzed pathologies. Our findings did not concur with the fourth and fifth systematic reviews,^9,10^ probably because of methodological differences. In comparison with placebo, an increased risk of adverse events and of withdrawals due to adverse events was observed in our study. Two of the reviews^8,10^ showed an increased risk of adverse events with cannabinoids, with one review^8^ specifically describing short-term and serious adverse events. However, these studies did not focus on MS.

Clear methodological differences exist among our systematic review and the ones published so far. The standardization conducted in our study allows comparison among different types of results that cannot be reliably compared otherwise. Furthermore, the high heterogeneity among the clinical assessment tools has been overcome by pooling those effect sizes evaluating the same outcome within the same study, avoiding both the exclusion of the studies where no coincidence between the clinical measures existed, as well as the risk of bias due to the inclusion of 1 unique clinical tool for analysis. Additionally, we included a specific tolerability analysis for the treatment of MS symptoms with cannabinoids.

### Limitations and Strengths

Limitations of our study include the small number of studies included; differences in the length of treatment, particularly in tolerability calculations; inclusion of crossover studies as parallel design; calculations made on the basis of an ITT principle by data extrapolation, which may have provoked bias in our results, although ITT analysis is the standard for medication evaluation; and publication bias. Another potential limitation is that blinding procedures can be affected in studies with drugs with such large difficulties in masking and blinding. Consequently, a large allocation-dependent placebo effect can be expected. This is particularly evident in the study with 2 phases in which the responders in the first phase were selected for the second one.^37^ In addition, most of the studies included were funded by the pharmaceutical industry, especially for nabiximols. As explained in the Results section, the exclusion of these studies had an impact on the results on subjective spasticity. In the interpretation of trends favoring experimental or control treatments, difficult decisions arose in some cases owing to the different forms of exposure across the studies.

Our study had strengths as well. The sensitivity analysis showed no relevant differences affecting the results obtained. We can thus consider our results to have a high level of certainty. Results in overall secondary calculations sustained the methods used. In addition, differing assessment tools were combined to evaluate a common outcome, considering the existence of procedural differences among tools. Nevertheless, caution was taken in the selection of tools with minimum differences. The combination maintained all the information provided by the studies and avoided a possibly subjective bias when selecting only 1 of the tools.

Shortcomings exist with respect to research into the efficacy of cannabinoids in the treatment of MS. The quantity of available studies is limited. However, they can be considered safe drugs, with no serious complications regarding withdrawal syndromes or drug dependence effects.

When comparing the efficacy of cannabinoids with other treatments for spasticity, such as baclofen or differing intrathecal doses of corticosteroids, in the Modified Ashworth scale, baclofen reduced the scores by a mean difference of 0.58^44^ and corticosteroids by 0.78.^45^ Cannabinoids (nabiximols) reduced spasticity in the same scale with a mean difference ranging from 0.1^37^ to 3.3.^35^ The risks and invasiveness of baclofen and corticosteroids should be considered.

In the case of bladder dysfunction, anticholinergic agents are the most common medication for this condition. Solifenacin, 5 mg, and oxybutynin, 15 mg, reduce the number of incontinence episodes per 24 hours by 1.03 and 2.41 vs placebo, respectively, in patients with MS and spinal cord injury,^46^ whereas injectable onabotulinumtoxinA, 300 U, reduces the same variable by 1.43.^47^ Cannabinoids vs placebo reduce the number of daily urge incontinence episodes by 0.21 (CE) and 0.16 (dronabinol)^27^ and reduce daily incontinence episodes by 0.11 (nabiximols).^36^ In comparison, cannabinoids show better tolerability than anticholinergics and less invasiveness than onabotulinumtoxinA.

As for pain, painful conditions are handled with drugs such as anticonvulsants, nonsteroidal anti-inflammatory agents, and corticosteroids. Nevertheless, management of pain in MS remains controversial and underresearched. Studies do not demonstrate clear efficacy of any treatment above others, whereas adverse events should be taken into consideration.^48,49,50^

There is no evidence of studies that evaluate the efficacy of cannabinoids vs other treatments in MS. Research into the possible combinations of cannabinoids and other therapies, therefore, might bring about greater synergy benefits than in an individual form.^51,52^

## Conclusions

Cannabinoids produce a limited and mild reduction of subjective spasticity, pain, and bladder dysfunction in patients with MS, but no changes in objectively measured spasticity. They can be considered safe drugs, as the analysis of serious adverse events did not show statistical significance, although the total number of adverse events is higher than in placebo for the treatment of symptoms in patients with MS.

## Notes

Supplement. **eReferences.** Full-text Records Excluded From Eligibility **eTable 1.** Characteristics of the Included Studies **eTable 2.** Summary of the Selected Clinical Assessment Tools **eTable 3.** Sensitivity Analysis Results for Efficacy Outcomes **eTable 4.** Sensitivity Analysis Results for Tolerability Outcomes **eFigure 1.** Risk of Bias Summary of the Included Studies **eFigure 2.** Risk of Bias Graph of the Included Studies **eFigure 3.** Funnel Plots for Efficacy Outcomes **eFigure 4.** Funnel Plots for Tolerability Outcomes
