# Supplementary material for: Assessment of Efficacy and Tolerability of Medicinal Cannabinoids in Patients With Multiple Sclerosis: A Systematic Review and Meta-analysis
Source: JAMA Netw Open. 2018 Oct 12;1(6):e183485. doi: 10.1001/jamanetworkopen.2018.3485 (PMC6324456; doi:10.1001/jamanetworkopen.2018.3485)
Supplement: Supplement. — eReferences. Full-text Records Excluded From Eligibility eTable 1. Characteristics of the Included Studies eTable 2. Summary of the Selected Clinical Assessment Tools eTable 3. Sensitivity Analysis Results for Efficacy Outcomes eTable 4. Sensitivity Analysis Results for Tolerability Outcomes eFigure 1. Risk of Bias Summary of the Included Studies eFigure 2. Risk of Bias Graph of the Included Studies eFigure 3. Funnel Plots for Efficacy Outcomes eFigure 4. Funnel Plots for Tolerability Outcomes [file jamanetwopen-1-e183485-s001.pdf]

Supplementary Online Content

Torres-Moreno MC, Papaseit E, Torrens M, Farré M. Assessment of efficacy and tolerability of medicinal cannabinoids in patients with multiple sclerosis: a systematic review and meta-analysis. *JAMA Netw Open*. 2018;1(6):e183485. doi:10.1001/jamanetworkopen.2018.3485

eReferences. Full-text Records Excluded From Eligibility

eTable 1. Characteristics of the Included Studies

eTable 2. Summary of the Selected Clinical Assessment Tools

eTable 3. Sensitivity Analysis Results for Efficacy Outcomes

eTable 4. Sensitivity Analysis Results for Tolerability Outcomes

eFigure 1. Risk of Bias Summary of the Included Studies

eFigure 2. Risk of Bias Graph of the Included Studies

eFigure 3. Funnel Plots for Efficacy Outcomes

eFigure 4. Funnel Plots for Tolerability Outcomes

This supplementary material has been provided by the authors to give readers additional information about their work.

1. Supplementary eReferences

eReferences 1: Full-text Records Excluded From Eligibility

e1. Svendsen KB, Jensen TS, Bach FW. [Effect of the synthetic cannabinoid dronabinol on central pain in patients with multiple sclerosis—secondary publication]. *Ugeskr Laeger*. 2005;167(25-31):2772-2774.

e2. Zajicek J, Ball S, Wright D, et al. Effect of dronabinol on progression in progressive multiple sclerosis (CUPID): a randomised, placebo-controlled trial. *Lancet Neurol*. 2013;12(9):857-865. doi:10.1016/S1474-4422(13)70159-5.

e3. Conte A, Bettolo CM, Onesti E, et al. Cannabinoid-induced effects on the nociceptive system: a neurophysiological study in patients with secondary progressive multiple sclerosis. *Eur J Pain*. 2009;13(5):472-477. doi:10.1016/j.ejpain.2008.05.014.

e4. Petro DJ, Ellenberger C. Treatment of human spasticity with delta 9-tetrahydrocannabinol. *J Clin Pharmacol*. 1981;21(8-9 Suppl):413S-416S.

e5. Clifford DB. Tetrahydrocannabinol for tremor in multiple sclerosis. *Ann Neurol*. 1983;13(6):669-671. doi:10.1002/ana.410130616.

e6. Ungerleider JT, Andyrsiak T, Fairbanks L, Ellison GW, Myers LW. Delta-9-THC in the treatment of spasticity associated with multiple sclerosis. *Adv Alcohol Subst Abuse*. 1987;7(1):39-50.

e7. Greenberg HS, Werness SA, Pugh JE, Andrus RO, Anderson DJ, Domino EF. Short-term effects of smoking marijuana on balance in patients with multiple sclerosis and normal volunteers. *Clin Pharmacol Ther*. 1994;55(3):324-328.

e8. Corey-Bloom J, Wolfson T, Gamst A, et al. Smoked cannabis for spasticity in multiple sclerosis: a randomized, placebo-controlled trial. *CMAJ*. 2012;184(10):1143-1150. doi:10.1503/cmaj.110837.

e9. Notcutt W, Langford R, Davies P, Ratcliffe S, Potts R. A placebo-controlled, parallel-group, randomized withdrawal study of subjects with symptoms of spasticity due to multiple sclerosis who are receiving long-term Sativex® (nabiximols). *Mult Scler*. 2012;18(2):219-228. doi:10.1177/1352458511419700.

e10. Zajicek JP, Sanders HP, Wright DE, et al. Cannabinoids in multiple sclerosis (CAMS) study: safety and efficacy data for 12 months follow up. *J Neurol Neurosurg Psychiatry*. 2005;76(12):1664-1669. doi:10.1136/jnnp.2005.070136.

e11. Serpell MG, Notcutt W, Collin C. Sativex long-term use: an open-label trial in patients with spasticity due to multiple sclerosis. *J Neurol*. 2013;260(1):285-295. doi:10.1007/s00415-012-6634-z.

e12. Rog DJ, Nurmikko TJ, Young CA. Oromucosal delta9-tetrahydrocannabinol/cannabidiol for neuropathic pain associated with multiple sclerosis: an uncontrolled, open-label, 2-year extension trial. *Clin Ther*. 2007;29(9):2068-2079. doi:10.1016/j.clinthera.2007.09.013.

e13. Wade DT, Makela PM, House H, Bateman C, Robson P. Long-term use of a cannabis-based medicine in the treatment of spasticity and other symptoms in multiple sclerosis. *Mult Scler*. 2006;12(5):639-645.

e14. Brady CM, DasGupta R, Dalton C, Wiseman OJ, Berkley KJ, Fowler CJ. An open-label pilot study of cannabis-based extracts for bladder dysfunction in advanced multiple sclerosis. *Mult Scler*. 2004;10(4):425-433.

e15. Russo M, Calabrò RS, Naro A, et al. Sativex in the management of multiple sclerosis-related spasticity: role of the corticospinal modulation. *Neural Plast*. 2015;2015:656582. doi:10.1155/2015/656582.

e16. Centonze D, Mori F, Koch G, et al. Lack of effect of cannabis-based treatment on clinical and laboratory measures in multiple sclerosis. *Neurol Sci*. 2009;30(6):531-534. doi:10.1007/s10072-009-0136-5.

e17. Russo M, Naro A, Leo A, et al. Evaluating Sativex® in Neuropathic Pain Management: A Clinical and Neurophysiological Assessment in Multiple Sclerosis. *Pain Med*. 2016;17(6):1145-1154. doi:10.1093/pm/pnv080.

© 2018 Torres-Moreno MC et al. *JAMA Network Open.*

e18. Wade DT, Robson P, House H, Makela P, Aram J. A preliminary controlled study to determine whether whole-plant cannabis extracts can improve intractable neurogenic symptoms. *Clin Rehabil*. 2003;17(1):21-29.

e19. Notcutt W, Price M, Miller R, et al. Initial experiences with medicinal extracts of cannabis for chronic pain: results from 34 "N of 1" studies. *Anaesthesia*. 2004;59(5):440-452. doi:10.1111/j.1365-2044.2004.03674.x.

e20. Wissel J, Haydn T, Müller J, et al. Low dose treatment with the synthetic cannabinoid Nabilone significantly reduces spasticity-related pain : a double-blind placebo-controlled cross-over trial. *J Neurol*. 2006;253(10):1337-1341. doi:10.1007/s00415-006-0218-8.

e21. Fox P, Bain PG, Glickman S, Carroll C, Zajicek J. The effect of cannabis on tremor in patients with multiple sclerosis. *Neurology*. 2004;62(7):1105-1109.

e22. Kurzthaler I, Bodner T, Kemmler G, et al. The effect of nabilone on neuropsychological functions related to driving ability: an extended case series. *Hum Psychopharmacol*. 2005;20(4):291-293. doi:10.1002/hup.688.

© 2018 Torres-Moreno MC et al. *JAMA Network Open.*

2. Supplementary eTables

eTable 1: Characteristics of the included studies

Data are mean (SD), N/n (%). Percentages may not add to 100% due to rounding. *Estimated by the authors of this systematic review from study data. †Interpreted by the authors of this systematic review based on study data. ‡ Computed only for the serious adverse events analysis, so not included in the total adverse events or in any other kind of adverse event analyses since only appeared in one of the included studies. DD = duration of disease. CBD = cannabidiol. CE = *Cannabis sativa* plant extract. IQR = interquartile range. ITT = intention-to-treat. MS = multiple sclerosis. N/n = no. of participants. PP = per-protocol. PPMS = primary progressive MS. PRMS = progressive relapsing MS. RCT = randomized clinical trial. RRMS = relapsing-remitting MS. SD = standard deviation. SPMS = secondary progressive MS. THC = δ-9-tetrahydrocannabinol. **Assessment:** 9-HPT = 9-Hole Peg Test. ADL = Activities of Daily Living. AMIPB = Adult Memory and Information Processing Battery. AS = Ashworth Scale. BDI = Beck Depression Inventory. BI = Barthel Index. BPI-SF = Brief Pain Inventory-Short Form. CRS = Category Rating Scale. C-SSRS = Columbia-Suicide Severity Rating Scale. DS of the WAIS-R= Digit Span of the Wechsler Adult Intelligence Scale-Revised. EDSS = Expanded Disability Status Scale. EQ-5D = European Quality of Life-5 Dimensions. FIM = Functional Independence Measure. fMRI = Functional Magnetic Resonance Imaging. FSS = Fatigue Severity Scale. GHQ-28 = General Health Questionnaire-28. GHQ-30 = General Health Questionnaire-30. GIC = Global Impression of Change. GNDS = Guy's Neurological Disability Scale. GSI = General Symptomatic Index. HADS = Hospital Anxiety and Depression Scale. HR-QoL = Health-Related Quality of Life. MAS = Modified Ashworth Scale. MI = Motricity Index. MRI = Magnetic Resonance Imaging. MSFC = Multiple Sclerosis Functional Composite Scale. MSIS-29 = Multiple Sclerosis Impact Scale-29. MSQoL-54 = Multiple Sclerosis Quality of Life-54. MSSS-88 = Multiple Sclerosis Spasticity Scale-88. MSWS-12 = Multiple Sclerosis Walking Scale-12. NNH = Number Needed to Harm. NNT = Number Needed to Treat. NOS = Not Otherwise Specified. NPS = Neuropathic Pain Scale. NRS = Numerical Rating Scale. PASAT = Paced Auditory Serial Addition Test. PDI = Pain Disability Index. PSQI = Pittsburgh Sleep Quality Index. PSS = Primary Symptom Score. QoL = Quality of Life. RMI = Rivermead Mobility Index. SAS = Self-rating Anxiety Scale. SCL-90-R = Symptom Checklist-90 Revised. SDMT = Symbol Digit Modalities Test. SF-36 = Short Form-36 Health Survey/Short Form questionnaire-36 items. SF-36 (PH) = Short Form questionnaire-36 items version 2 (physical health subscale). SF-MPQ = Short-Form McGill Pain Questionnaire. SPART = 10/36 Spatial Recall Test. SRT = Selective Reminding Test. TMS = Transcranial Magnetic Stimulation. T10-MW = Timed 10-m walk. T25-FW = Timed 25-foot walk. UIE = Urge incontinence episode. UKNDS = United Kingdom Neurological Disability Scale. VAS = Visual Analog Scale. WLG = Word List Generation.

		**Interventions**			
**Study**	**All participants**	**THC/CBD (CE or nabiximols)**	**THC (dronabinol or nabilone)**	**Placebo**	**Conclusions**
**Killestein 2002**[25]					
Design	Progressive MS patients with spasticity<br>Setting not specified<br>RCT, placebo-controlled<br>Double-blind<br>Crossover<br>20 weeks (4-week/intervention, 4-week washout between treatment periods)<br>ITT analysis	Oral CE caps.:<br>(2.5 mg THC + 20 to 30% CBD of standardized THC content)/cap.	Oral dronabinol caps.:<br>2.5 mg THC/cap.	Placebo caps.	No reduction of spasticity.
		Dose = 2-4 caps./day<br>(5-10 mg THC + approx. 1.25-2.5 mg CBD)	Dose = 2-4 caps./day<br>(5-10 mg THC)	Dose = 2-4 caps./day	
$N/n_{initial}$	16	16	16	16	
Sex	..	..	..	..	
Subtype of MS					
SPMS	10 (63%)	..	..	..	
PPMS	6 (38%)	..	..	..	
Mean age (SD) years	46.0 (7.90)	..	..	..	
Mean DD (SD) years	15.0 (10.70)	..	..	..	
Mean EDSS (SD)	6.2 (1.20)	..	..	..	
N/n_{final}	16	16	16	16	
Assessment					
Efficacy:<br>*Included in our analysis*: AS.<br>*Excluded from our analysis*: 9-HPT; Concentration VAS; EDSS, and EDSS-Brainstem functional systems domain; Fatigue VAS; HR-QoL Questionnaire, and HR-QoL-Psychological status domain; Micturition VAS; Mood VAS; MSFC; MS-specific FSS; Pain VAS; PASAT; SF-36, and SF-36-Mental health subscale; Spasticity VAS; Subject´s Global Impression VAS; T25-FW; Tremor VAS; Vision VAS; and Walking VAS.<br>*Tolerability:*<br>*Included in our analysis*: Ataxia; dizziness; dry mouth; headache; increased spasticity; somnolence; and withdrawals due to adverse events.<br>*Excluded from our analysis*: Acute psychosis; emotional lability; and others.					
					(continue)

© 2018 Torres-Moreno MC et al. *JAMA Network Open.*

eTable 1: Characteristics of the included studies *(continued)*					
		**Interventions**			
**Study**	**All participants**	**THC/CBD (CE or nabiximols)**	**THC (dronabinol or nabilone)**	**Placebo**	**Conclusions**
**Zajicek 2003[26]/Freeman 2006[27]** (2 publications with same cohort of study) *(continue)*					
**Zajicek 2003[26]**					
Design	MS patients with spasticity<br>Multicentric (UK)<br>RCT, placebo-controlled<br>Double-blind<br>Parallel<br>15 weeks (13-week treatment, 1-week dose withdrawal, 1-week off trial medication)<br>ITT analysis	Oral CE caps.:<br>(2.5 mg THC + 1.25 mg CBD)/cap.	Oral dronabinol caps.:<br>2.5 mg THC/cap.	Placebo caps.	No beneficial effect on spasticity.
		Dose = 4-10 caps./day<br>(10-25 mg THC + 5-12.5 mg CBD)<br>Body weight based	Dose = 4-10 caps./day<br>(10-25 mg THC)<br>Body weight based	Dose = 4-10 caps./day<br><br>Body weight based	
		Mean dose (SD) =<br>5.42 (2.11) caps./day<br>(13.56 mg THC + 6.78 mg CBD)*	Mean dose (SD) =<br>5.47 (2.08) caps./day<br>(13.67 mg THC)*	Mean dose (SD) =<br>6.24 (1.71) caps./day*	
$N/n_{initial}$	630	211	206	213	
Sex					
M	217 (34%)	76 (36%)	63 (31%)	78 (37%)	
F	413 (66%)	135 (64%)	143 (69%)	135 (63%)	
Subtype of MS					
RRMS	33 (5%)	6 (3%)	14 (7%)	13 (6%)	
SPMS	452 (72%)	152 (72%)	149 (72%)	151 (71%)	
PPMS	145 (23%)	53 (25%)	43 (21%)	49 (23%)	
Mean age (SD) years	..	50.5 (7.60)	50.2 (8.20)	50.9 (7.60)	
Mean AS (SD)	..	21.8 (8.70)	22.6 (10.10)	21.4 (8.50)	
EDSS, number of patients (range)	..	209 (6.4-7.7)*	203 (6.4-7.7)*	212 (6.3-7.6)*	
N/n_{final}	611 (97%)	207 (98%)	197 (96%)	207 (97%)	
Assessment					
Efficacy:<br>*Included in our analysis*: AS; Bladder symptoms Questionnaire; Pain CRS; Pain Questionnaire; and Spasticity Questionnaire.<br>*Excluded from our analysis*: BI; Depression CRS; EDSS; Energy (amount of energy) CRS; GHQ-30; Irritability CRS; Muscle spasms CRS; RMI; Shake/tremor (tremor) CRS; Sleep quality CRS; Spasticity (Muscle stiffness) CRS; T10-MW; Tiredness CRS; Tremor Questionnaire; and UKNDS.<br>*Tolerability:*<br>*Included in our analysis*: Abdominal pain of unknown cause; active duodenal ulcer and helicobacter pylori; back pain; chest infection/urinary tract infection; collapse of unknown cause; collapse/bradycardia; constipation; death; deep-vein thrombosis; diarrhoea; dizzy or lightheadedness; dry mouth; fall at home; grand mal seizures; infection; minor cerebrovascular event; MS relapse or possible relapse; numbness or paraesthesia; pain; pneumonia; possible transient ischaemic attack/syncope; sleep; tremor or lack coordination; urinary tract infection; urinary tract infection/relapse; viral gastroenteritis; vision; and withdrawals due to adverse events.<br>*Excluded from our analysis*: Bladder; blocked/insertion of suprapubic catheter‡; cellulitis of leg/diarrhoea and vomiting‡; chronic pleural effusion‡; depression or anxiety; disease progression (not relapse) ‡; dizziness (inappropriate SAE report); emergency hip replacement‡; gastrointestinal tract; improvements in symptoms; increased appetite; miscellaneous; pneumonia and renal stones‡; spasms or stiffness; urinary tract infection/diarrhoea and vomiting‡; and weakness or reduced mobility.					
					(continue)

© 2018 Torres-Moreno MC et al. *JAMA Network Open.*

**eTable 1: Characteristics of the included studies** *(continued)*					
		**Interventions**			
**Study**	**All participants**	**THC/CBD (CE or nabiximols)**	**THC (dronabinol or nabilone)**	**Placebo**	**Conclusions**
**Zajicek 2003[26]/Freeman 2006[27]** (2 publications with same cohort of study) *(continued)*					
**Freeman 2006[27]** (same as Zajicek 2003,[26] unless specified)					
Design	Patients recruited to Zajicek 2003[26] (CAMS study), excepting of those with a permanent catheter.	..	..	..	Clinical effect on incontinence episodes.
$N/n_{initial}$ (% with respect to Zajicek 2003[26] initial data)	522 (83%)	181 (86%)	174 (84%)	167 (78%)	
N/n_{final} (% with respect to Zajicek 2003[26] initial data)	255 (40%)	88 (42%)	86 (42%)	81 (38%)	
Assessment					
Efficacy:<br>*Included in our analysis*: UIEs Diary.<br>*Excluded from our analysis*: King's Health Questionnaire; Pad test (weight); Post-void residual; and Voiding cystometry.<br>*Tolerability:*<br>*Included in our analysis*: Urinary tract infections (included in Zajicek 2003[26], but not specified as urinary tract infections), and worsening of detrusor-sphincter-dyssynergia (D-S-D) and urinary retention.<br>*Excluded from our analysis*: None.					
					(continue)

© 2018 Torres-Moreno MC et al. *JAMA Network Open.*

eTable 1: Characteristics of the included studies *(continued)*

		Interventions			
**Study**	**All participants**	**THC/CBD (CE or nabiximols)**	**THC (dronabinol or nabilone)**	**Placebo**	**Conclusions**
**Svendsen 2004**[28]					
Design	MS patients with central neuropathic pain<br>Unicentric (Denmark)<br>RCT, placebo-controlled<br>Double-blind<br>Crossover<br>9 weeks (3-week/intervention, 3-week washout between treatment periods)<br>ITT analysis	..	Oral dronabinol caps.:<br>2.5 mg THC/cap.	Placebo caps.	Clinical effect on central pain.
			Dose = 2.5 mg-10 mg THC/day (1-4 caps.)	Dose = 2.5 mg-10 mg/day	
			Mean dose (range) =<br>3.1 [2.7-3.6] caps./day<br>(7.75 [6.75-9.00] mg THC)	Mean dose (range) =<br>3.3 [2.8-3.6] caps./day<br>(8.25 [7.00-22.50] mg)	
$N/n_{initial}$	24	..	24	24	
Sex					
M	10 (42%)	..	..	..	
F	14 (58%)	..	..	..	
Subtype of MS					
RRMS	9 (38%)	..	..	..	
SPMS	9 (38%)	..	..	..	
PPMS	6 (25%)	..	..	..	
Median age (range)	50 (23-55)	..	..	..	
Median DD (range)	7.0 (0.3-25.0)	..	..	..	
Median EDSS (range)	6.0 (2.5-6.5)	..	..	..	
Median Pain intensity NRS (range)	5.5 (3.0-8.0)	..	..	..	
N/n_{final}	24	..	24	24	
Assessment					
Efficacy:<br>*Included in our analysis*: Pain-relief NRS Diary; Radiating pain NRS Diary; SF-36-Bodily pain subscale; and Spontaneous pain intensity NRS Diary.<br>*Excluded from our analysis*: 50% Pain relief; EDSS; NNT (50% reduction in central pain), Use of scape medication Diary; Quantitative sensory testing; SF-36-General health, SF-36-Mental health, SF-36-Physical functioning, SF-36-Role emotional, SF-36-Role physical, SF-36-Social functioning, and SF-36-Vitality subscales; and Treatment preference.<br>*Tolerability:*<br>*Included in our analysis*: Abdominal pain; anorexia; balance difficulty; diplopia; distortion of wrist; dizziness or lightheadedness; euphoria; fatigue; feeling of drunkenness; fever; headache; migraine; mouth dryness; muscle weakness; myalgia; nausea; palpitations; speech disorders; upper airway infection; weight decrease; and withdrawals due to adverse events.<br>*Excluded from our analysis*: Chills; hot flushes; hyperactivity; limb heaviness; multiple sclerosis aggravated (one patient admitted to the hospital); nervousness; sleep difficulty; tenderness in nose; and tiredness or drowsiness.					
					(continue)

© 2018 Torres-Moreno MC et al. *JAMA Network Open.*

eTable 1: Characteristics of the included studies *(continued)*

		Interventions			
**Study**	**All participants**	**THC/CBD (CE or nabiximols)**	**THC (dronabinol or nabilone)**	**Placebo**	**Conclusions**
**Vaney 2004**[29]					
Design	MS patients with spasticity<br>Unicentric (Switzerland)<br>RCT, placebo-controlled<br>Double-blind<br>Crossover<br>4 weeks (2-week cannabinoids treatment, 1-week placebo, 3-day washout between/after interventions)<br>ITT/PP analyses	Oral CE caps.:<br>(2.5 mg THC + 0.9 mg CBD)/cap.	..	Placebo caps.	Reduction of spasm frequency and increase of mobility.
		Dose = 6-12 caps./day<br>(15-30 mg THC + 5.4-10.8 mg CBD)		Dose = 6-12 caps./day	
		Mean dose (SD) =<br>(17.99 [7.63] mg THC + 6.48 [2.75] mg CBD)/day<br>(7.20 caps.)*		Mean dose not specified	
$N/n_{initial}$	57	57	..	57	
Sex					
M	28 (49%)	..	..	..	
F	29 (51%)	..	..	..	
Subtype of MS					
RRMS	2 (4%)	..	..	..	
SPMS	26 (46%)	..	..	..	
PPMS	29 (51%)	..	..	..	
Mean age (SD) years	54.9 (10.00)	..	..	..	
Mean DD (SD) years	17.0 (8.40)	..	..	..	
Median EDSS (range)	7.0 (6.00)	..	..	..	
Mean AS (SD)	12.5 (6.20)	..	..	..	
N/n_{final}	50 (88%)	50 (88%)	..	50 (88%)	
Assessment					
Efficacy:<br>*Included in our analysis*: AS; and Micturition problems Questionnaire Diary.<br>*Excluded from our analysis*: 9-HPT; DS of the WAIS-R; EDSS; Falling asleep fast Diary; FIM; PASAT; RMI; Spasm-frequency Diary; T10-MW; Tremor Questionnaire Diary; and Waking up again Diary.<br>*Tolerability:*<br>*Included in our analysis*: Blurred vision; constipation; dizziness; dry mouth; euphoria, "high"; headache; nausea, feeling sick; pain in extremities; palpitations; sleepiness; sleeplessness; tremor or shakes; and withdrawals due to adverse events.<br>*Excluded from our analysis*: Cannabinoid toxicity Questionnaire Diary-Likert Scale, difficulty concentrating; fall; feeling aggressive; flu-like symptoms; and inadequate laughing.					
					(continue)

© 2018 Torres-Moreno MC et al. *JAMA Network Open.*

eTable 1: Characteristics of the included studies *(continued)*

		Interventions			
**Study**	**All participants**	**THC/CBD (CE or nabiximols)**	**THC (dronabinol or nabilone)**	**Placebo**	**Conclusions**
**Wade 2004**[30]					
Design<br>Registered in *ClinicalTrials.gov* (NCT01610700, study results posted)	MS patients with spasticity, spasms, bladder problems, tremor, or pain (not musculoskeletal)<br>Multicentric (UK)<br>RCT, placebo-controlled<br>Double-blind<br>Parallel<br>6 weeks (6-week treatment)<br>PP analysis	Oromucosal CE (nabiximols): (2.7 mg THC + 2.5 mg CBD)/spray	..	Placebo spray	Reduction of spasticity.
		Dose = 1-48 sprays/day (2.7-129.6 mg THC + 2.5-120 mg CBD<br>Self-titrated dose		Dose =1-48 sprays/day<br>Self-titrated dose	
		Mean dose (SD) = 12.37 (6.05) sprays/day (33.40 mg THC + 30.93 mg CBD)*		Mean dose (SD) = 18.87 (6.17) sprays/day*	
$N/n_{initial}$	160	80	..	80	
Sex					
M	61 (38%)	33 (41%)	..	28 (35%)	
F	99 (62%)	47 (59%)	..	52 (65%)	
Subtype of MS	..	..	..	..	
Mean age (SD) years	50.7 (9.32)	51.0 (9.36)	..	50.4 (9.33)	
Mean MAS (SD)	..	5.0 (3.70)	..	4.6 (4.40)	
N/n_{final}	154 (96%)	77 (96%)	..	77 (96%)	
Assessment					
Efficacy:<br>*Included in our analysis*: Bladder (Bladder Problems) VAS; Bladder Questionnaire (Bladder Control Test); Bladder VAS Diary; MAS; Pain VAS Diary; Pain VAS; Spasticity VAS Diary; and Spasticity VAS.<br>*Excluded from our analysis*: 9-HPT; AMIPB; BDI-II; BI; Care-giver Strain Index Score; Feeling upon waking VAS; FSS; GHQ-28; GNDS (UKNDS); How much sleep (Sleep Amount) VAS; PSS (Composite Primary Impairment VAS); Quality of sleep (Sleep Quality) VAS; Reading Visual Acuity Test; RMI; Short Orientation-Memory-Concentration Test; Spasm Frequency VAS Diary; Spasm severity VAS Diary; Spasms (Muscle Spasm) VAS; Subject Global Opinion of Effect on Multiple Sclerosis; Summed Symptom Score; T10-MW (Ten-metre Mobility Score); Tremor ADL Scale; Tremor VAS Diary; and Tremor VAS.<br>*Tolerability:*<br>*Included in our analysis*: Appendicitis; application site discomfort; application site pain; application site reaction NOS; diarrhoea; disorientation; disturbance in attention; dizziness; dry mouth; euphoric mood; fatigue; feeling drunk; headache; hypoaesthesia; lower respiratory tract infection NOS; mouth ulceration; muscle spasms; muscle weakness NOS; nausea; oral discomfort; oral pain; pain in limb; respiratory distress; sepsis NOS; somnolence; upper respiratory tract infection NOS; urinary tract infection NOS; vertigo; and withdrawals due to adverse events.<br>*Excluded from our analysis*: Arthritis NOS‡; cough; and Feeling of intoxication VAS Diary.					
					(continue)

© 2018 Torres-Moreno MC et al. *JAMA Network Open.*

eTable 1: Characteristics of the included studies *(continued)*					
		Interventions			
**Study**	**All participants**	**THC/CBD (CE or nabiximols)**	**THC (dronabinol or nabilone)**	**Placebo**	**Conclusions**
**Rog 2005**[31]					
Design<br>Registered in *ClinicalTrials.gov* (NCT01604265, study results posted)	MS patients with central neuropathic pain<br>Unicentric(UK)<br>RCT, placebo-controlled<br>Double-blind<br>Parallel<br>5 weeks (1-week run in, 4-week treatment)<br>ITT analysis	Oromucosal CE (nabiximols): (2.7 mg THC + 2.5 mg CBD)/spray	..	Placebo	Reduction of central neuropathic pain and sleep disturbance.
		Dose = 1-48 sprays/day (2.7-129.6 mg THC + 2.5-120 mg CBD)<br>Self-titrated dose		Dose =1-48 sprays/day<br>Self-titrated dose	
		Mean dose (SD) = 9.6 (6.1) sprays/day (week 4) (25.92 mg THC + 24.00 mg CBD)		Mean dose (SD) = 19.1 (12.9) sprays/day (week 4)	
$N/n_{initial}$	66	34	..	32	
Sex					
M	14 (21%)	6 (18%)	..	8 (25%)	
F	52 (79%)	28 (82%)	..	24 (75%)	
Subtype of MS					
RRMS	23 (35%)	..	..	..	
SPMS	33 (50%)	..	..	..	
PPMS	9 (14%)	..	..	..	
Benign MS	1 (2%)	..	..	..	
Mean age (SD) years	49.2 (8.32)	50.3 (6.70)	..	48.1 (9.73)	
Mean DD (SD) years	11.6 (7.70)	10.4 (7.30)	..	12.8 (8.10)	
Mean EDSS (SD)	5.9 (1.30)	6.0 (1.10)	..	5.8 (1.50)	
Mean Pain NRS (SD)	6.5 (1.60)	6.5 (1.60)	..	6.4 (1.70)	
N/n_{final}	64 (97%)	32 (94%)	..	32 (100%)	
Assessment					
Efficacy:<br>*Included in our analysis*: NPS; Pain (central neuropathic pain) NRS; and Sleep disturbance (due to neuropathic pain) NRS.<br>*Excluded from our analysis*: GNDS (UKNDS); HADS-Anxiety, and HADS-Depression items; MSFC; NNT (50% reduction in central pain); PASAT; Patient´s (Subject) GIC; SDMT; SPART; SRT; and WLG.<br>*Tolerability:*<br>*Included in our* analysis: Application site burning; back pain; breast pain; confusion; diarrhoea; diplopia; disorientation; dissociation; disturbance in attention; dizziness; dry mouth; dyspepsia; dyspnea; euphoria; falls; fatigue; feeling abnormal; feeling drunk; gamma-glutamyltransferase increased; glossodynia; hallucination; headache; hypoaesthesia; intoxication (agitation, tachycardia and hypertension)†; ligament sprain; logorrhea; migraine NOS; mouth ulceration; nasopharyngitis; nausea; oral pain; otitis media NOS; paraesthesia; paranoia; paranoid ideation; pharyngitis; rash NOS; sinusitis NOS; skin irritation; somnolence; urinary tract infection NOS; vomiting; weakness; white blood cell count increased; and withdrawals due to adverse events.<br>*Excluded from our analysis*: Chest discomfort; crying; hoarseness; Intoxication Levels VAS; low mood; NNH; thirst; and throat irritation.					
					(continue)

© 2018 Torres-Moreno MC et al. *JAMA Network Open.*

eTable 1: Characteristics of the included studies *(continued)*

		Interventions			
**Study**	**All participants**	**THC/CBD (CE or nabiximols)**	**THC (dronabinol or nabilone)**	**Placebo**	**Conclusions**
**Collin 2007**[32]					
Design<br>Registered in *ClinicalTrials.gov* (NCT00711646, study results posted)	MS patients with spasticity, failed to gain adequate relief using current therapy<br>Multicentric (UK and Romania)<br>RCT, placebo-controlled<br>Double-blind<br>Parallel<br>6 weeks (6-week treatment)<br>ITT/PP analyses	Oromucosal CE (nabiximols): (2.7 mg THC + 2.5 mg CBD)/spray<br>Dose = 1-48 sprays/day (2.7-129.6 mg THC + 2.5-120 mg CBD)<br>Self-titrated dose<br>Mean dose (SD) = 9.4 (6.4) sprays/day (25.38 mg THC + 23.50 mg CBD)	..	Placebo<br>Dose =1-48 sprays/day<br>Self-titrated dose<br>Mean dose (SD) = 14.7 (8.4) sprays/day	Reduction of spasticity.
$N/n_{initial}$	189	124	..	65	
Sex					
M	75 (40%)	44 (35%)	..	31 (48%)	
F	114 (60%)	80 (65%),	..	34 (52%)	
Mean age (SD) years	49.1 (9.90)	49.7 (10.20)	..	47.8 (9.50)	
Mean DD (SD) years	12.6 (SD not specified)	13.6 (8.60)	..	12.2 (7.70)	
N/n_{final}	174 (92%)	112 (90%)	..	62 (95%)	
Assessment					
Efficacy:<br>*Included in our analysis*: AS; and Spasticity NRS Diary.<br>*Excluded from our analysis*: MI (arms), and MI (legs) items; Patient´s GIC; Spasm-frequency NRS Diary; Spasticity NRS 30% responder analysis; and Spasticity NRS 50% responder analysis.<br>*Tolerability:*<br>*Included in our analysis*: Appendicitis; balance impaired; Bartholin's abscess; confusion; constipation; depressed mood; diarrhoea; disorientation; disturbance in attention; dizziness; dry mouth; dysgeusia; euphoric mood; fatigue; headache; lower respiratory tract infection NOS; nausea; oral pain; pain in limb; pancreatic carcinoma NOS; pulmonary embolism; somnolence; urinary tract infection; urinary tract infection NOS; vision blurred; vomiting; weakness; and withdrawals due to adverse events.<br>*Excluded from our analysis*: Mobility decreased‡; Intoxication NRS Diary; and urinary incontinence aggravated‡.					
					(continue)

© 2018 Torres-Moreno MC et al. *JAMA Network Open.*

eTable 1: Characteristics of the included studies *(continued)*

		Interventions			
**Study**	**All participants**	**THC/CBD (CE or nabiximols)**	**THC (dronabinol or nabilone)**	**Placebo**	**Conclusions**
**Aragona 2009[33]/Tomassini 2014[34]** (2 publications with same cohort of study) *(continue)*					
**Aragona 2009[33]**					
Design	SPMS patients with spasticity<br>Unicentric (Italy)<br>RCT, placebo-controlled<br>Double-blind<br>Crossover<br>10 weeks (3-week/intervention, 2-week washout between/after treatment periods)<br>ITT/PP analyses	Oromucosal CE (nabiximols): (2.7 mg THC + 2.5 mg CBD)/spray<br>Dose (specified in Tomassini 2014[34])<br>Mean dose (SD) = 8.20 (3.15) sprays/day (22.14 mg THC + 20.50 mg CBD)	..	Placebo<br>Dose (specified in Tomassini 2014[34])<br>Mean dose (SD) = 15.16 (4.51) sprays/day	No psychopathology induction and no cognition impairment.
$N/n_{initial}$ (% with respect to Tomassini 2014[34] initial data)	17 (94%)	17	..	17	
Sex					
M	6 (35%)	..	..	..	
F	11 (65%)	..	..	..	
Subtype of MS					
SPMS	17	..	..	..	
Mean age (SD) years	49.8 (6.64)	..	..	..	
Mean DD (SD) years	20.76 (8.42)	..	..	..	
Mean EDSS (SD)	6.1 (0.30)	..	..	..	
N/n_{final} (% with respect to Tomassini 2014[34] initial data)	17 (94%)	17	..	17	
Assessment					
Efficacy:<br>*Included in our analysis*: None.<br>*Excluded from our analysis*: 9-HPT; FSS; HR-QoL VAS (EQ-5D Health status VAS); MSIS-29 Physical, and MSIS-29 Psychological items; PASAT; SAS; SCL-90-R-Aggressive behaviour, SCL-90-R-Anxiety, SCL-90-R-Depression, SCL-90-R-Obsessive-compulsive features, SCL-90-R-Paranoiac tendencies, SCL-90-R-Phobic anxiety, SCL-90-R-Psychotic symptoms, SCL-90-R-Sensitivity, and SCL-90-R-Somatized anxiety dimensions; SCL-90-R-GSI item; and T25-FW. Plasma measurements.<br>*Tolerability:*<br>*Included in our analysis*: Craving†; depression; dizziness and vertigo; drowsiness and/or slower thinking; euphoria; fatigue; headache; intoxication (transient mental confusion with temporal and spatial disorientation, tachycardia, increased blood pressure, and mydriasis)†;lower limb weakness; mouth dryness or burning; nausea and vomiting; secondary depression; tremor; and withdrawals due to adverse events.<br>*Excluded from our analysis*: None.					
					(continue)

© 2018 Torres-Moreno MC et al. *JAMA Network Open.*

eTable 1: Characteristics of the included studies *(continued)*

		Interventions			
**Study**	**All participants**	**THC/CBD (CE or nabiximols)**	**THC (dronabinol or nabilone)**	**Placebo**	**Conclusions**
**Aragona 2009**[33]**/Tomassini 2014**[34] (2 publications with same cohort of study) *(continued)*					
**Tomassini 2014**[34] (same as Aragona 2009,[33] unless specified)					
Design<br>Registered in *ClinicalTrials.gov* (NCT00202423, no study results posted)	..	Dose = 1-48 sprays/day (2.7-129.6 mg THC + 2.5-120 mg CBD)<br>Self-titrated dose<br>Median dose (range) = 7.4 (2.7-12.5) sprays/day (19.98 mg THC + 18.50 mg CBD)	..	Dose = 1-48 sprays/day<br>Self-titrated dose<br>Median dose (range) = 16.1 (6.7-26) sprays/day	No significant benefits on spasticity.<br>No change in fMRI motor-evoked brain activation. No difference in intracortical and spinal motor excitability.
N/n$_{initial}$	18	18	..	18	
Sex					
M	6 (33%)	..	..	..	
F	12 (67%)	..	..	..	
Subtype of MS					
SPMS	18	..	..	..	
Median age (range) years	51 (37-59)	..	..	..	
Median DD (range) years	21.5 (5-35)	..	..	..	
Median EDSS (range)	6.0 (6.0-6.5)	..	..	..	
Median AS (range)	12 (4-22)	..	..	..	
Median Spasticity NRS (range)	7 (3-9)	..	..	..	
N/n$_{final}$	18	18	..	18	
Assessment					
Efficacy:<br>*Included in our analysis*: AS; and Spasticity NRS.<br>*Excluded from our analysis*: AS (upper/lower extremities) 30% responders; and Spasticity NRS 30% responders. Neurophysiological assessment (fMRI, H-reflex, and TMS); and plasma measurements.<br>*Tolerability:* None.					
					(continue)

© 2018 Torres-Moreno MC et al. *JAMA Network Open.*

eTable 1: Characteristics of the included studies *(continued)*					
		**Interventions**			
**Study**	**All participants**	**THC/CBD (CE or nabiximols)**	**THC (dronabinol or nabilone)**	**Placebo**	**Conclusions**
**Collin 2010**[35]					
Design<br>Registered in *ClinicalTrials.gov* (NCT01599234, study results posted)	MS patients with spasticity, not wholly relieved with current therapy<br>Multicentric (UK and Czech Republic)<br>RCT, placebo-controlled<br>Double-blind<br>Parallel<br>15 weeks (1-week baseline, 14-week treatment)<br>ITT/PP analyses	Oromucosal CE (nabiximols): (2.7 mg THC + 2.5 mg CBD)/spray<br>Dose = 1-24 sprays/day (2.7-64.8 mg THC + 2.5-60 mg CBD)<br>Self-titrated dose<br>Mean dose (range) = 8.5 (1-22) sprays/day (22.95 mg THC + 21.25 mg CBD)	..	Placebo<br>Dose = 1-24 sprays/day<br>Self-titrated dose<br>Mean dose (range) = 15.4 (2-23) sprays/day	No significant improvement in spasticity NRS.
$N/n_{initial}$	337	167	..	170	
Sex					
M	130 (39 %)	61 (37%)	..	69 (41%)	
F	207 (61%)	106 (63%)	..	101 (59%)	
Mean age (SD) years	47.5 (9.61)	48.0 (10.06)	..	47.1 (9.15)	
Mean DD (SD) years	15.2 (8.41)	14.4 (8.29)	..	16.0 (8.48)	
Mean EDSS (SD)	6.0 (1.53)	6.0 (1.56)	..	6.0 (1.50)	
N/n_{final}	305 (91%)	150 (90%)	..	155 (91%)	
Assessment					
Efficacy:<br>*Included in our analysis*: Bladder symptoms NRS; MAS; Pain NRS; Sleep quality (due to spasticity) NRS; and Spasticity NRS Diary.<br>*Excluded from our analysis*: BI; Caregiver´s GIC; EQ-5D Health state index, and EQ-5D Health status VAS items; Fatigue NRS; MSQoL-54-Mental health, and MSQoL-54-Physical health composites; Number of subjects with a 50% or greater improvement in mean Spasticity NRS; Spasm severity NRS; Spasticity NRS 30% responder analysis; Spasticity NRS Time to 30% response; T10-MW; and Tremor NRS.<br>*Tolerability:*<br>*Included in our analysis*: Anxiety; asthenia; back pain; burns third degree; confusional state; constipation; death; dehydration; depressed mood; depression; diarrhoea; disorientation; dissociation; disturbance in attention; dizziness; drug dependence; dry mouth; dysarthria; dysgeusia; dyspepsia; epilepsy; erysipelas; euphoric mood; fall; fatigue; feeling abnormal; foot fracture; gastrointestinal carcinoma with liver metastases; hallucination; headache; infections and infestations; insomnia; metastatic oesophageal carcinoma; MS relapse; muscle spasms; muscle spasticity; nausea; orchitis; paranoia; peripheral ischaemia; phlebothrombosis; sepsis; sleep attacks; somnolence; suicidal ideation; tetany; urinary tract infection; urinary retention; urinary tract infection NOS; vertigo; vomiting; withdrawal syndrome (aggression, agitation, delusions, irritability, insomnia and muscle spasms)†; and withdrawals due to adverse events.<br>*Excluded from our analysis*: Apathy; decubitus ulcer‡; haemoptysis‡; malaise; road traffic accident‡; and worsened depression‡.					
					(continue)

© 2018 Torres-Moreno MC et al. *JAMA Network Open.*

eTable 1: Characteristics of the included studies *(continued)*

		Interventions			
**Study**	**All participants**	**THC/CBD (CE or nabiximols)**	**THC (dronabinol or nabilone)**	**Placebo**	**Conclusions**
**Kavia 2010**[36]					
Design<br>Registered in *ClinicalTrials.gov* (NCT00678795, study results posted)	MS patients with overactive bladder (OAB), failed to respond adequately to first-line therapies<br>Multicentric (UK, Belgium and Romania)<br>RCT, placebo-controlled<br>Double-blind<br>Parallel<br>10 weeks (2-week baseline, 8-week treatment)<br>ITT/PP analyses	Oromucosal CE (nabiximols): (2.7 mg THC + 2.5 mg CBD)/spray	..	Placebo	Non-statistical significance reduction in Number of episodes of incontinence. Some significant positive effects on other bladder symptoms.
		Dose = 1-48 sprays/day (2.7-129.6 mg THC + 2.5-120 mg CBD)<br>Self-titrated dose		Dose = 1-48 sprays/day<br>Self-titrated dose	
		Mean dose (median) = 8.91 (7.19) sprays/day (24.06 mg THC + 22.28 mg CBD)		Mean dose (median) = 17.05 (14.22) sprays/day	
N/$n_{initial}$	135	67	..	68	
Sex					
M	37 (27%)	15 (22%)	..	22 (32%)	
F	98 (73%)	52 (78%)	..	46 (68%)	
Mean age (SD) years	47.7 (10.31)	48.6 (9.31)	..	46.8 (11.20)	
Episodes of Incontinence/day (number of patients)	..	1.8 (n = 63)	..	2.1 (n = 66)	
Episodes of Nocturia/day (number of patients)	..	1.6 (n = 63)	..	1.5 (n = 66)	
N/n_{final}	118 (87%)	56 (84%)	..	62 (91%)	
Assessment					
Efficacy:<br>*Included in our analysis*: Bladder symptom severity (Overall Bladder Condition, OBC) NRS; Daily number of incontinence episodes Diary; Incontinence pad weight; Incontinence QoL Questionnaire; Nocturia episodes Diary (per day); Number Daytime voids Diary (per day); Number of incontinence pads used Diary (per day); and Void urgency episodes Diary (per day).<br>*Excluded from our analysis*: Cystometric capacity (Voiding cystometry); Patient´s GIC; Post-void residual volume; Responder analysis of the frequency of urgency; Total number of voids Diary (per 24 h); and Volume voided (per 24h).<br>*Tolerability:*<br>*Included in our analysis*: Abdominal pain upper; anorexia; application site pain; balance impaired; chest pain; confusion; constipation; cystitis; dehydration; diarrhoea; disorientation; dissociation; disturbance in attention; dizziness; fatigue; feeling abnormal; feeling drunk; haemorrhagic cystitis; headache; influenza; intoxication (shaking, coordination problems and severe absence)†; memory impairment; MS relapse; nasopharyngitis; nausea; neck pain; paraesthesia; pharyngitis; pharyngitis viral NOS; pyrexia; somnolence; toothache; urinary tract infection; vertigo; vomiting; weakness; and withdrawals due to adverse events.<br>*Excluded from our analysis*: Intoxication NRS; and sweating increased.					
					(continue)

© 2018 Torres-Moreno MC et al. *JAMA Network Open.*

eTable 1: Characteristics of the included studies *(continued)*

		Interventions			
**Study**	**All participants**	**THC/CBD (CE or nabiximols)**	**THC (dronabinol or nabilone)**	**Placebo**	**Conclusions**
**Novotna 2011**[37]					
Design (enriched study design)<br>Registered in *ClinicalTrials.gov* (NCT00681538, study results posted)	MS patients with spasticity, not wholly relieved with current antispasticity medication, and at least a 20% reduction in mean Spasticity NRS score after 4 weeks of the previous single-blind phase A treatment (responders)<br>Multicentric (UK, Spain, Poland, Czech Republic and Italy)<br>RCT, placebo-controlled<br>Double-blind (phase B)<br>Parallel<br>12 weeks (12-week treatment)<br>ITT/PP analyses	Oromucosal CE (nabiximols): (2.7 mg THC + 2.5 mg CBD)/spray<br>Dose = 1-12 sprays/day (2.7-32.4 mg THC + 2.5-30 mg CBD)<br>Self-titrated dose<br>Mean dose (SD) = 8.3 (2.43) sprays/day (22.41 mg THC + 20.75 mg CBD)	..	Placebo<br>Dose = 1-12 sprays/day<br>Self-titrated dose<br>Mean dose (SD) = 8.9 (2.31) sprays/day	Significant reduction in spasticity.
N/n$_{initial}$	241	124	..	117	
Sex					
M	96 (40%)	52 (42%)	..	44 (38%)	
F	145 (60%)	72 (58%)	..	73 (62%)	
Mean age (SD) years	48.6 (9.33)	49.1 (9.09)	..	48.1 (9.59)	
Mean DD (SD) years	12.6 (7.88)	13.3 (8.29)	..	11.8 (7.38)	
Mean EDSS (SD)	6.0 (1.45)	6.5 (1.46)	..	6.0 (1.44)	
Mean spasticity NRS (SD)	3.9 (1.51)	3.9 (1.49)	..	3.9 (1.55)	
N/n$_{final}$	224 (93%)	109 (88%)	..	115 (98%)	
Assessment					
Efficacy:<br>*Included in our analysis*: MAS; SF-36-Bodily pain subscale; Sleep Disruption (due to spasticity) NRS; and Spasticity NRS.<br>*Excluded from our analysis*: BDI-II; BI; Carer GIC; Clinician GIC; EQ-5D Health state index; EQ-5D Health status VAS items; MI (arms), and MI (legs) items; Physician GIC; SF-36-General health, SF-36-Mental health, SF-36-Physical functioning, SF-36-Role emotional, SF-36-Role physical, SF-36-Social functioning, and SF-36-Vitality subscales; Spasm frequency NRS; Spasticity NRS 30% responders; Spasticity NRS 50% responders; Subject´s GIC; and T10-MW.<br>*Tolerability:*<br>*Included in our analysis*: Abdominal pain (upper); back pain; balance disorder; bronchopneumonia; diarrhoea; dizziness; dry mouth; euphoric mood; fatigue; headache; infections and infestations; MS relapse; muscle spasms; muscle spasticity; nasopharyngitis; nausea; pain in extremity; septic shock; somnolence; suicidal ideation; urinary tract infection; urosepsis; vertigo; and withdrawals due to adverse events.<br>*Excluded from our analysis*: Pregnancy‡.					
					(continue)

© 2018 Torres-Moreno MC et al. *JAMA Network Open.*

eTable 1: Characteristics of the included studies *(continued)*					
		**Interventions**			
**Study**	**All participants**	**THC/CBD (CE or nabiximols)**	**THC (dronabinol or nabilone)**	**Placebo**	**Conclusions**
**Zajicek 2012**[38]					
Design<br>Registered in *ClinicalTrials.gov* (NCT00552604, no study results posted)	MS patients with muscle stiffness<br>Multicentric (UK)<br>RCT, placebo-controlled<br>Double-blind<br>Parallel<br>14 weeks (2-week screening, 12-week treatment)<br>ITT analysis	Oral CE caps.:<br>(2.5 mg THC + 1.25 mg CBD)/caps.<br>Dose = 2-10 caps./day<br>(5 mg-25 mg THC + 2.5 mg-12.5 mg CBD)<br>Self-titrated dose<br>Mean dose (SD) =<br>7.81 (2.75) caps./day (end of titration period)<br>(19.52 mg THC + 9.76 mg CBD)*<br>Mean dose (SD) =<br>6.81 (2.99) caps./day (end of study period)<br>(17.02 mg THC + 8.51 mg CBD)*	..	Placebo caps.<br>Dose = 2-10 caps./day<br>(5 mg-25 mg/day)<br>Self-titrated dose<br>Mean dose (SD) =<br>9.60 (1.27) caps./day (end of titration period)<br>(24.00 mg)*<br>Mean dose (SD) =<br>9.36 (1.51) caps./day (end of study period)<br>(23.40 mg)*	Effect in the treatment of muscle stiffness.
$N/n_{initial}$	277	143	..	134	
Sex					
M	102 (37%)	55 (38%)	..	47 (35%)	
F	175 (63%)	88 (62%)	..	87 (65 %)	
Subtype of MS					
RRMS	21 (8%)	13 (9%)	..	8 (6%)	
SPMS	190 (69%)	96 (67%)	..	94 (70%)	
PPMS	66 (24%)	34 (24%)	..	32 (24%)	
Mean age (SD) years	..	51.9 (7.70)	..	52.0 (7.90)	
Mean DD (SD) years	..	14.5 (9.50)	..	15.1 (8.40)	
N/n_{final}	224 (81%)	109 (76%)	..	115 (86%)	
Assessment					
Efficacy:<br>*Included in our analysis:* Body pain CRS; and MSSS-88-Ability to walk, MSSS-88-ADL/Daily activities, MSSS-88-Body movement, MSSS-88-Feelings, MSSS-88-Muscle spasms, MSSS-88-Muscle stiffness, MSSS-88-Pain and discomfort, and MSSS-88-Social functioning subscales.<br>*Excluded from our analysis*: EDSS; MSIS-29 Physical impact, and MSIS-29 Psychological impact items; MSWS-12; Muscle spasms CRS; Muscle stiffness CRS; and Sleep quality (Quality of sleep) CRS.<br>*Tolerability:*<br>*Included in our analysis*: Asthenia; dizziness; dry mouth; fatigue; headache; urinary tract infection; and withdrawals due to adverse events.<br>*Excluded from our analysis*: Balance disorder; confusional state; diarrhoea; disorientation; disturbance in attention; fall; feeling abnormal; head injury; interstitial lung; nausea; pain in extremities; and somnolence.					
					(continue)

© 2018 Torres-Moreno MC et al. *JAMA Network Open.*

eTable 1: Characteristics of the included studies *(continued)*					
		**Interventions**			
**Study**	**All participants**	**THC/CBD (CE or nabiximols)**	**THC (dronabinol or nabilone)**	**Placebo**	**Conclusions**
**Langford 2013**[39]					
Design<br>Registered in *ClinicalTrials.gov* (NCT00391079, study results posted)	MS patients with central neuropathic pain<br>Multicentric (UK, Czech Republic, Canada, Spain and France)<br>RCT, placebo-controlled<br>Double-blind (phase A)<br>Parallel<br>15 weeks (1-week baseline, 14-week treatment)<br>ITT/PP analyses	Oromucosal CE (nabiximols): (2.7 mg THC + 2.5 mg CBD)/spray<br>Dose = 8-12 sprays/day (21.6-32.4 mg THC + 20-30 mg CBD)<br>Self-titrated dose<br>Mean dose (SD) = 8.8 (3.87) sprays/day (23.76 mg THC + 22.00 mg CBD)	..	Placebo<br>Dose = 8-12 sprays/day<br>Self-titrated dose<br>Mean dose (SD) = 11.1 (4.6) sprays/day	No statistical significant reduction of pain.
$N/n_{initial}$	339	167	..	172	
Sex					
M	109 (32%)	54 (32 %)	..	55 (32 %)	
F	230 (68%)	113 (68 %)	..	117 (68 %)	
Subtype of MS					
RRMS	157 (46%)	80 (48%)	..	77 (45%)	
SPMS	136 (40%)	65 (39%)	..	71 (41%)	
PPMS	40 (12%)	18 (11%)	..	22 (13%)	
PRMS	6 (2%)	4 (2%)	..	2 (1%)	
Mean age (SD) years	48.97 (10.47)	48.42 (10.43)	..	49.51 (10.50)	
Mean DD (SD) years	11.99 (8.26)	11.42 (8.00)	..	12.53 (8.50)	
Mean Pain NRS (SD)	6.58 (1.32)	6.55 (1.35)	..	6.61 (1.29)	
N/n_{final}	297 (88%)	141 (84%)	..	156 (91%)	
Assessment					
Efficacy:<br>*Included in our analysis*: Bladder symptoms NRS; BPI-SF; NPS; Pain (due to MS) NRS; PDI; SF-36-Bodily pain subscale; Sleep quality (Sleep Disruption, due to pain) NRS; Spasticity NRS; and Subjec´s GIC for pain.<br>*Excluded from our analysis*: Breakthrough analgesia; EQ-5D Health state index, and EQ-5D Health status VAS items; Fatigue NRS; Pain NRS 30% responder analysis; Pain NRS 50% responder analysis; SF-36-General health, SF-36-Mental health, SF-36-Physical functioning, SF-36-Role emotional, SF-36-Role physical, SF-36-Social functioning, and SF-36-Vitality subscales; Spasms severity NRS; and Tremor NRS.<br>*Tolerability:*<br>*Included in our analysis*: Balance disorder; constipation; depression; diarrhoea; disturbance in attention; dizziness; dry mouth; dysgeusia; fatigue; feeling abnormal; headache; hepatic enzyme increased; infections and infestations; memory impairment; muscular weakness; nausea; neuralgia; orthostatic hypotension; pain; pain in extremity; pharyngolaryngeal pain; somnolence; suicidal ideation; syncope; vertigo; vision blurred; vomiting; and withdrawals due to adverse events.<br>*Excluded from our analysis*: Motor dysfunction‡; and psychomotor skills impaired.					
					(continue)

© 2018 Torres-Moreno MC et al. *JAMA Network Open.*

**eTable 1: Characteristics of the included studies** *(continued)*					
		**Interventions**			
**Study**	**All participants**	**THC/CBD (CE or nabiximols)**	**THC (dronabinol or nabilone)**	**Placebo**	**Conclusions**
**Vachová 2014**[40]					
Design<br>Registered in *ClinicalTrials.gov* (NCT01964547, study results posted)	MS patients with spasticity, not wholly relieved with current anti-spasticity therapy<br>Multicentric (Czech Republic)<br>RCT, placebo-controlled<br>Double-blind<br>Parallel<br>50 weeks (48-week treatment, 2-week end of treatment follow-up period)<br>ITT/PP analyses	Oromucosal CE (nabiximols): (2.7 mg THC + 2.5 mg CBD)/spray<br>Dose = 1-12 sprays/day (2.7-32.4 mg THC + 2.5-30 mg CBD)<br>Self-titrated dose<br>Mean dose (SD) = 7.6 (3.1) sprays/day (first month) (20.52 mg THC + 19.00 mg CBD)<br>6.4 (3.1) sprays/day (last three months) (17.28 mg THC + 16.00 mg CBD)	..	Placebo<br>Dose = 1-12 sprays/day<br>Self-titrated dose<br>Mean dose (SD) = 9.5 (2.4/2.6).sprays/day (from first to last three months)	No association with cognitive decline or significant changes in mood (long-term treatment). No statistical significant difference in spasticity.
N/$n_{initial}$	121	62	..	59	
Sex					
M	45 (37%)	23 (37%)	..	22 (37%)	
F	76 (63%)	39 (63%)	..	37 (63%)	
Subtype of MS					
RRMS	59 (49%)	26 (42%)	..	33 (56%)	
SPMS	43 (36%)	24 (39%)	..	19 (32%)	
PPMS	16 (13%)	11 (18%)	..	5 (8%)	
PRMS	3 (2%)	1 (2%)	..	2 (3%)	
Mean age (SD) years	48.6 (9.64)	49.0 (8.95)	..	48.2 (10.38)	
Mean DD (SD) years	13.9 (8.55)	13.9 (8.09)	..	13.9 (9.08)	
Mean Spasticity NRS (SD)	6.7 (1.86)	6.7 (2.04)	..	6.7 (1.67)	
N/n_{final}	98 (81%)	50 (81%)	..	48 (81%)	
Assessment					
Efficacy:<br>*Included in our analysis*: MAS; and Subject´s GIC for spasticity.<br>*Excluded from our analysis*: BDI-II; Caregiver´s GIC; C-SSRS; Number of visits to a healthcare professional; PASAT; Physician´s GIC; and T10-MW.<br>*Tolerability:*<br>*Included in our analysis*: Acute myocardial infarction; anxiety disorder due to a general medical condition; application site discomfort; asthenia; back pain; bacterial infection; blood alkaline phosphatase increased; bronchitis; cerebellar ataxia; death; decreased appetite; dermatitis allergic; diarrhoea; disorientation; dizziness; drug withdrawal syndrome; dry mouth; dysarthria; euphoric mood; fatigue; foot fracture; forearm fracture; gingivitis; headache; herpes zoster; joint dislocation; ligament sprain; lower limb fracture; memory impairment; MS relapse; muscle spasms; muscle spasticity; nausea; neuralgia; oral mucosal erythema; oropharyngeal blistering; overdose; pain in extremity; paraesthesia; paraparesis; pneumonia; pyrexia; somnolence; subcutaneous abscess; suicidal ideation; tetany; thermal burn; tonsillitis; tremor; trigeminal neuralgia; upper limb fracture; upper respiratory tract infection; upper respiratory tract infection bacterial; urinary tract infection; vertigo; viral infection; visual impairment; vitamin D decreased; vomiting; weight decreased; and withdrawals due to adverse events.<br>*Excluded from our analysis*: Cognitive disorder; contusion; drug hypersensitivity; erectile dysfunction; face injury; inguinal hernia‡; lipoma excision; MS; procedural vomiting; radiculopathy; stupor; and tooth extraction.					
					(continue)

© 2018 Torres-Moreno MC et al. *JAMA Network Open.*

eTable 1: Characteristics of the included studies *(continued)*					
		Interventions			
**Study**	**All participants**	**THC/CBD (CE or nabiximols)**	**THC (dronabinol or nabilone)**	**Placebo**	**Conclusions**
**Turcotte 2015**[41]					
Design<br>Registered in *ClinicalTrials.gov* (NCT00480181, no study results posted)	RRMS patients with neuropathic pain, on a non-effective treatment with gabapentin at a stabilized dose of ≥1800 mg/day for at least 1 month<br>Unicentric (Canada)<br>RCT, placebo-controlled<br>Double-blind<br>Parallel<br>9 weeks (9-week treatment)<br>ITT/PP analyses	..	Oral nabilone caps.:<br>Available 0.5 or 1 mg THC/cap.<br>Dose = 1 (0.5 mg THC/caps.)-2 (1 mg THC/caps.) caps./day (0.5-2 mg THC)<br>Mean dose not specified	Placebo caps.<br>Dose = 1 (0.5 mg/caps.)-2 (1 mg /caps.) caps./day (0.5-2 mg)<br>Mean dose not specified	Nabilone as an adjunctive to gabapentin is an effective, well-tolerated combination for MS-induced neuropathic pain.
$N/n_{initial}$	15	..	8	7	
Sex					
M	2 (13%)	..	1 (13%)	1 (14%)	
F	13 (87%)	..	7 (88%)	6 (86%)	
Subtype of MS					
RRMS	15	..	..	..	
Mean age (SD) years	45.5 (10.84)	..	42.12 (11.20)	50.0 (8.48)	
Median DD (IQR) years	6.5 (5-8.75)	..	5.5 (4.5-7.25)	8 (6.25-9)	
Mean EDSS (SD)	2.82 (0.77)	..	2.56 (0.77)	3.17 (1.07)	
Mean Pain intensity (SD)	77.00 (14.04)	..	79.00 (13.76)	74.33 (13.99)	
Mean Pain impact (SD)	59.85 (23.47)	..	63.00 (19.23)	54.8 (30.86)	
N/n_{final}	14 (93%)	..	7 (88%)	7 (100%)	
Assessment					
Efficacy:<br>*Included in our analysis*: Impact of pain on daily activities VAS Diary (VAS_{impact}); Pain intensity VAS Diary (VAS_{pain}); Patient-rated GIC for neuropathic pain.<br>*Excluded from our analysis*: EDSS; SF-36; and SF-MPQ.<br>*Tolerability:*<br>*Included in our analysis*: Withdrawals due to adverse events.<br>*Excluded from our analysis*: Dizziness; drowsiness; dry mouth; and headache.					
					(continue)

© 2018 Torres-Moreno MC et al. *JAMA Network Open.*

eTable 1: Characteristics of the included studies *(continued)*					
		Interventions			
**Study**	**All participants**	**THC/CBD (CE or nabiximols)**	**THC (dronabinol or nabilone)**	**Placebo**	**Conclusions**
**Ball 2015[42]**					
Design	Progressive MS patients<br>Multicentric (UK)<br>RCT, placebo-controlled<br>Double-blind<br>Parallel<br>3 years (3-year treatment)<br>ITT/PP analyses	..	Oral THC caps. (dronabinol):<br>3.5 mg THC	Placebo caps.	No effect in slowing progression of MS.
			Dose = 2-8 caps./day<br>(7-28 mg THC)<br>Titrated against body weight and adverse effects	Dose = 2-8 caps./day<br><br>Titrated against body weight and adverse effects	
			Median dose = 4 (25th–75th percentiles 2–6) caps./day (final year of follow-up)<br>(14.00 mg THC)	Median dose = 6 (25th–75th percentiles 4–8) caps./day (final year of follow-up)	
$N/n_{initial}$	493	..	329	164	
Sex					
M	201 (41%)	..	133 (40%)	68 (41%)	
F	292 (59%)	..	196 (60%)	96 (59%)	
Subtype of MS					
SPMS	302 (61%)	..	203 (62%)	99 (60%)	
PPMS	191 (39%)	..	126 (38%)	65 (40%)	
Mean age (SD) years	52.19 (7.80)	..	52.29 (7.60)	51.97 (8.20)	
Mean EDSS (SD)	5.9 (0.69)	..	5.8 (0.69)	5.9 (0.67)	
N/n_{final}	415 (84%)	..	267 (81%)	148 (90%)	
Assessment					
Efficacy: (3 years, unless specified)<br>*Included in our analysis*: Bladder problems CRS; and MSSS-88-Ability to walk/Walking, MSSS-88-ADL/Daily activities, MSSS-88-Body movements, MSSS-88-Feelings, MSSS-88-Muscle spasms, MSSS-88-Muscle stiffness, MSSS-88-Pain and discomfort, and MSSS-88-Social functioning subscales.<br>*Excluded from our analysis*: 9-HPT (annual change); BDI-II; Co-ordination CRS; Depression CRS; EDSS (number of first progression events per patient-year); EQ-5D five dimensions questionnaire; Fatigue CRS; Forgetfulness CRS; Irritability CRS; MSFC; MSIS-29 Physical and MSIS-29 Psychological items; MSWS-12; PASAT (annual change); RMI (annual change); Sensory loss or numbness CRS; SF-36, SF-36 (PH) (annual change); T25-FW (annual change); and Tremor CRS. Neurophysiological measures (MRI).<br>*Tolerability:*<br>*Included in our analysis*: Death; dissociative and thinking or perception disorders; dizziness and lightheadedness; falls and injuries; fatigue and tiredness; infections (excluding urinary tract); joint disorders; mobility, balance, and coordination problems; mood disorders (depression); muscle disorders (weakness); musculoskeletal pain and aches; urinary tract infections; and withdrawals due to adverse events.<br>*Excluded from our analysis*: Admission to hospital‡; constipation, diarrhoea, or faecal incontinence; life-threatening or important medical event‡; and muscle disorders (spasticity, stiffness, spasms, or tremor).					
					(continue)

© 2018 Torres-Moreno MC et al. *JAMA Network Open.*

eTable 1: Characteristics of the included studies *(continued)*					
		**Interventions**			
**Study**	**All participants**	**THC/CBD (CE or nabiximols)**	**THC (dronabinol or nabilone)**	**Placebo**	**Conclusions**
**Leocani 2015[43]**					
Design<br>Registered in *ClinicalTrials.gov* (NCT01538225, no study results posted)	Progressive MS patients with lower limb spasticity, stable drug treatment not able to relieve symptoms as a whole<br>Unicentric (Italy)<br>RCT, placebo-controlled<br>Double-blind<br>Crossover<br>10 weeks (4-week/intervention, 2-week washout between treatment periods)<br>PP analysis	Oromucosal CE (nabiximols): (2.7 mg THC + 2.5 mg CBD)/spray<br>Dose = 1-12 sprays/day (2.7-32.4 mg THC + 2.5-30 mg CBD)<br>Self-titrated dose<br>Mean dose (SD) = 7 (3) sprays/day (18.90 mg THC + 17.50 mg CBD)	..	Placebo<br>Dose = 1-12 sprays/day<br>Self-titrated dose<br>Mean dose (SD) = 10 (3) sprays/day	Clinical benefit in objective lower limb spasticity. Lack of corresponding changes in corticospinal excitability and on the monosynaptic component of the stretch reflex.
N/$n_{initial}$	43	43	..	43	
Sex					
M	23 (53%)	..	..	..	
F	20 (47%)	..	..	..	
Subtype of MS					
Progressive MS	43	..	..	..	..
Mean age (SD) years	48 (8.00)	..	..	..	
Mean DD (SD) years	17.1 (8.40)	..	..	..	
Mean EDSS (SD)	5.5 (1.00)	..	..	..	
Mean MAS (SD), lower limbs	8.1 (3.90)				
Mean MAS (SD), total	9.3 (5.20)	..	..	..	
Mean Spasticity NRS (SD)	7.0 (1.50)	..	..	..	
N/n_{final}	34 (79%)	34 (79%)	..	34 (79%)	
Assessment					
Efficacy:<br>*Included in our analysis*: MAS (lower limbs, extreme outliner value retained); Pain NRS; and Spasticity NRS.<br>*Excluded from our analysis*: 9-HPT (dominant hand), and 9-HPT (non-dominant hand); FSS; MAS (lower limbs, extreme outliner value removed); MAS (upper limbs); MAS 20% responders; Sleep quality NRS (PSQI); Spasm frequency; Spasticity NRS 20% responders; and T10-MW. Neurophysiological assessment (H-reflex, and TMS).<br>*Tolerability:*<br>*Included in our analysis*: Dizziness; faringodynia; fever; hypotension; lower limb weakness; somnolence; subjective weakness; vertigo; and withdrawals due to adverse events.<br>*Excluded from our analysis*: Acute pancreatitis‡; and hypertension.					

**eTable 2: Summary of the selected clinical assessment tools**		
†Items, or some of them, composing the tool were included individually in the statistical analysis.<br>CRS = Category Rating Scale. GIC = Global Impression of Change. MS = multiple sclerosis. NRS = Numerical Rating Scale. QoL = quality of life. VAS = Visual Analog Scale.		
**Clinical assessment tool[Publication where analyzed]**	**Description**	**Evaluated outcomes**
Ashworth scale[25,26,29,32,34]	Scored by an observer. Measures passive resistance to movement during muscle stretching.	Spasticity (Ashworth)
Bladder (Bladder Problems) VAS[30]	Bladder problems.	Bladder dysfunction
Bladder problems CRS[42]	Bladder problems.	Bladder dysfunction
Bladder questionnaire (Bladder Control Test)[30]	Bladder symptoms and control, and effects on the patient's life.	Bladder dysfunction
Bladder symptom severity (Overall Bladder Condition, OBC) NRS[36]	Severity of urinary incontinence and general bladder symptoms.	Bladder dysfunction
Bladder symptoms questionnaire[26]	Overall effect of medication in bladder function.	Bladder dysfunction
Bladder symptoms NRS[35,39]	Bladder symptoms.	Bladder dysfunction
Bladder VAS Diary[30]	Severity of bladder symptoms.	Bladder dysfunction
Body pain CRS[38]	Pain.	Pain
Brief Pain Inventory-Short Form (BPI-SF)[39]	Rate pain and the degree to which pain interferes with activities.	Pain
Daily number of incontinence episodes Diary[36]	Incontinence episode frequency.	Bladder dysfunction
Impact of pain on daily activities VAS Diary (VAS_{impact})[41]	Impact of pain on daily activities.	Pain
Incontinence pad weight[36]	Weight of the incontinence pads used.	Bladder dysfunction
Incontinence QoL Questionnaire[36]	Impact of lower urinary tract symptoms on patient´s quality of life.	Bladder dysfunction
Micturition problems questionnaire Diary[29]	Micturition.	Bladder dysfunction
Modified Ashworth scale[30,35,37,40,43]	Modified version of the Ashworth scale adding a 1+ grade for resistance throughout the reminder (less than half) of the range of movement.	Spasticity (Ashworth)
Multiple Sclerosis Spasticity Scale-88 (MSSS-88)†	Self-reported assessment of impact of spasticity in MS patients in 8 areas (ability to walk, activities of daily living, body movement, feelings, muscle spasms, muscle stiffness, pain and discomfort, and social functioning).	..
MSSS-88-Ability to walk/Walking subscale[38,42]	Effect of spasticity on walking mobility.	Spasticity (subjective)
MSSS-88-Activities of daily living/Daily activities subscale[38,42]	Effect of spasticity on daily activities.	Spasticity (subjective)
MSSS-88-Body movement subscale[38,42]	Effect of spasticity on body movements.	Spasticity (subjective)

© 2018 Torres-Moreno MC et al. *JAMA Network Open.*

eTable 2: Summary of the selected clinical assessment tools *(continued)*		
**Clinical assessment tool** Publication where analyzed	**Description**	**Evaluated outcomes**
MSSS-88-Feelings subscale[38,42]	Effect of spasticity on feelings.	Spasticity (subjective)
MSSS-88-Muscle spasms subscale[38,42]	Muscle spasms due to spasticity.	Spasticity (subjective)
MSSS-88-Muscle stiffness subscale[38,42]	Muscle stiffness due to spasticity.	Spasticity (subjective)
MSSS-88-Pain and discomfort subscale[38,42]	Pain and discomfort due to spasticity.	Pain<br>Spasticity (subjective)
MSSS-88-Social functioning subscale[38,42]	Effect of spasticity on social functioning.	Spasticity (subjective)
Neuropathic Pain Scale (NPS)[31,39]	Level of neuropathic pain.	Pain
Nocturia episodes Diary (per day)[36]	Instances of nocturia, and the time that each took place.	Bladder dysfunction
Number Daytime voids Diary (per day)[36]	Number of voids.	Bladder dysfunction
Number of incontinence pads used Diary (per day)[36]	Number of incontinence pads used.	Bladder dysfunction
Pain (central neuropathic pain) NRS[31]	Central neuropathic pain.	Pain
Pain (pain due to MS) NRS[39]	Level of pain due to MS.	Pain
Pain disability index (PDI)[39]	Degree to which aspects of patient´s life are disrupted by chronic pain.	Pain
Pain intensity VAS Diary (VAS_{pain})[41]	Pain intensity.	Pain
Pain questionnaire[26]	Overall effect of medication in pain.	Pain
Pain CRS[26]	Pain.	Pain
Pain NRS[35,43]	Pain.	Pain
Pain-relief NRS Diary[28]	Measurement of the level of relief in pain.	Pain
Pain VAS[30]	Pain.	Pain
		(continue)

© 2018 Torres-Moreno MC et al. *JAMA Network Open.*

eTable 2: Summary of the selected clinical assessment tools *(continued)*		
**Clinical assessment tool**[Publication where analyzed]	**Description**	**Evaluated outcomes**
Pain VAS Diary[30]	Severity of pain.	Pain
Patient-rated GIC for neuropathic pain[41]	Overall patient-perceived effect of treatment on their pain levels.	Pain
Radiating pain NRS Diary [28]	Pain.	Pain
Short Form-36 Health Survey/Short Form questionnaire-36 items (SF-36)†	Self-rated health status questionnaire. Two summary scales (physical component summary and the mental component summary) are generated from 8 subscales (physical functioning, role limitations due to physical problems, bodily pain, general health perceptions, vitality, social functioning, role-limitations due to emotional problems, and mental health).	..
SF-36-Bodily pain subscale[28,37,39]	Pain level and interference with normal work.	Pain
Sleep Disruption (due to spasticity) NRS [37]	Sleep disruption due to spasticity.	Spasticity (subjective)
Sleep disturbance (due to neuropathic pain) NRS[31]	Sleep disruption due to neuropathic pain.	Pain
Sleep quality (Sleep Disruption, due to pain) NRS[39]	Sleep disruption due to pain.	Pain
Sleep quality (due to spasticity) NRS[35]	Sleep disruption due to spasticity.	Spasticity (subjective)
Spasticity questionnaire[26]	Overall effect of medication in spasticity.	Spasticity (subjective)
Spasticity NRS[34,37,39,43]	Spasticity.	Spasticity (subjective)
Spasticity NRS Diary[32,35]	Spasticity.	Spasticity (subjective)
Spasticity VAS[30]	Spasticity.	Spasticity (subjective)
Spasticity VAS Diary[30]	Spasticity.	Spasticity (subjective)
Spontaneous pain intensity NRS Diary[28]	Pain.	Pain
Subject´s GIC (SGIC) for pain[39]	Status of pain due to multiple sclerosis since entry into the study.	Pain
Subject´s GIC (SGIC) for spasticity[40]	Change in spasticity since immediately before receiving the first dose of study treatment.	Spasticity (subjective)
Urge Incontinence Episodes Diary[27]	Number of incontinence episodes.	Bladder dysfunction
Void urgency episodes Diary (per day)[36]	Instances of urgency, and the time that each took place.	Bladder dysfunction

© 2018 Torres-Moreno MC et al. *JAMA Network Open.*

eTable 3: Sensitivity analysis results for efficacy outcomes

Results obtained performing meta-analysis using the inverse of variance method and the random effects model (except SA1, where fixed effects model was used) on an ITT basis. Sensitivity analysis was conducted using the same statistical methods than the main analysis, but modifying the parameter specified in each of the five performed analyses (SA1 to SA5): Main analysis (all studies included[25–43]). SA1: Use of the fixed-effects model instead of random effects (all studies included[25–43]). SA2: Exclusion of studies with crossover design (5 studies, 6 publications, excluded[25,28,29,33,34,43]). SA3: Exclusion of studies with a sample size of 50 patients or fewer (5 studies, 6 publications, excluded[25,28,33,34,41,43]). SA4: Exclusion of studies with a length of treatment of 4 weeks or less (6 studies, 7 publications, excluded[25,28,29,31,33,34,43]). SA5: Exclusion of studies with a high risk of bias (7 studies, 9 publications, excluded[25–27,33,34,37,41–43]). Not estimable: Not available studies in the intervention group. Results with statistical significance shown in bold type.
SA = Sensitivity analysis. SMD = *Hedges´g* standardized mean difference.

		**SMD (95% CI)**					
**Outcome**	**Intervention**	**Main analysis**	**SA1**	**SA2**	**SA3**	**SA4**	**SA5**
Spasticity (Ashworth)	Cannabis extract	0.01 (-0.18 to 0.20)	0.01 (-0.18 to 0.20)	-0.05 (-0.29 to 0.18)	0.05 (-0.22 to 0.31)	-0.05 (-0.29 to 0.18)	0.23 (-0.14 to 0.60)
	Nabiximols	-0.11 (-0.22 to 0.01)	-0.11 (-0.22 to 0.01)	-0.10 (-0.22 to 0.02)	-0.10 (-0.22 to 0.02)	-0.10 (-0.22 to 0.02)	-0.06 (-0.20 to 0.08)
	Dronabinol	-0.16 (-0.38 to 0.07)	-0.16 (-0.38 to 0.07)	-0.16 (-0.39 to 0.07)	-0.16 (-0.39 to 0.07)	-0.16 (-0.39 to 0.07)	Not estimable
	Cannabinoids	-0.09 (-0.18 to 0.0027)	-0.09 (-0.18 to 0.0027)	**-0.10 (-0.20 to -0.0035)**	-0.08 (-0.17 to 0.02)	**-0.10 (-0.20 to -0.0035)**	-0.03 (-0.17 to 0.12)
Spasticity (subjective)	Cannabis extract	**-0.27 (-0.44 to -0.09)**	**-0.27 (-0.44 to -0.09)**	**-0.27 (-0.44 to -0.09)**	**-0.27 (-0.44 to -0.09)**	**-0.27 (-0.44 to -0.09)**	-0.20 (-0.44 to 0.04)
	Nabiximols	**-0.29 (-0.47 to -0.12)**	**-0.26 (-0.36 to -0.15)**	**-0.32 (-0.53 to -0.11)**	**-0.32 (-0.53 to -0.11)**	**-0.32 (-0.53 to -0.11)**	**-0.27 (-0.49 to -0.06)**
	Dronabinol	-0.13 (-0.46 to 0.20)	-0.09 (-0.24 to 0.06)	-0.13 (-0.46 to 0.20)	-0.13 (-0.46 to 0.20)	-0.13 (-0.46 to 0.20)	Not estimable
	Cannabinoids	**-0.25 (-0.38 to -0.13)**	**-0.22 (-0.29 to -0.14)**	**-0.26 (-0.40 to -0.13)**	**-0.26 (-0.40 to -0.13)**	**-0.26 (-0.40 to -0.13)**	**-0.25 (-0.43 to -0.08)**
Pain	Cannabis extract	**-0.33 (-0.50 to -0.16)**	**-0.33 (-0.50 to -0.16)**	**-0.33 (-0.50 to -0.16)**	**-0.33 (-0.50 to -0.16)**	**-0.33 (-0.50 to -0.16)**	**-0.29 (-0.53 to -0.05)**
	Nabiximols	-0.07 (-0.26 to 0.12)	-0.07 (-0.18 to 0.05)	-0.12 (-0.29 to 0.05)	-0.12 (-0.29 to 0.05)	-0.07 (-0.19 to 0.05)	-0.09 (-0.29 to 0.11)
	Dronabinol	-0.23 (-0.55 to 0.09)	**-0.15 (-0.29 to -0.0041)**	-0.17 (-0.53 to 0.20)	-0.17 (-0.53 to 0.20)	-0.17 (-0.53 to 0.20)	-0.50 (-1.08 to 0.08)
	Nabilone	**-1.40 (-2.78 to -0.03)**	**-1.40 (-2.78 to -0.03)**	**-1.40 (-2.78 to -0.03)**	Not estimable	**-1.40 (-2.78 to -0.03)**	Not estimable
	Cannabinoids	**-0.17 (-0.31 to -0.03)**	**-0.15 (-0.23 to -0.07)**	**-0.19 (-0.33 to -0.06)**	**-0.18 (-0.31 to -0.05)**	**-0.17 (-0.29 to -0.04)**	-0.17 (-0.34 to 0.01)

© 2018 Torres-Moreno MC et al. *JAMA Network Open.*

**eTable 3: Sensitivity analysis results for efficacy outcomes** *(continued)*							
		**SMD (95% CI)**					
**Outcome**	**Intervention**	**Main analysis**	**SA1**	**SA2**	**SA3**	**SA4**	**SA5**
Bladder dysfunction	Cannabis extract	**-0.29 (-0.50 to -0.09**)	**-0.29 (-0.50 to -0.09**)	**-0.27 (-0.52 to -0.02**)	**-0.29 (-0.50 to -0.09**)	**-0.27 (-0.52 to -0.02**)	-0.34 (-0.71 to 0.03)
	Nabiximols	-0.07 (-0.22 to 0.08)	-0.06 (-0.18 to 0.07)	-0.07 (-0.22 to 0.08)	-0.07 (-0.22 to 0.08)	-0.07 (-0.22 to 0.08)	-0.07 (-0.22 to 0.08)
	Dronabinol	-0.06 (-0.27 to 0.16)	-0.04 (-0.19 to 0.11)	-0.06 (-0.27 to 0.16)	-0.06 (-0.27 to 0.16)	-0.06 (-0.27 to 0.16)	Not estimable
	Cannabinoids	**-0.11 (-0.22 to -0.0008**)	**-0.09 (-0.18 to -0.01**)	-0.09 (-0.20 to 0.02)	**-0.11 (-0.22 to -0.0008**)	-0.09 (-0.20 to 0.02)	-0.11 (-0.26 to 0.04)

© 2018 Torres-Moreno MC et al. *JAMA Network Open.*

eTable 4: Sensitivity analysis results for tolerability outcomes

Results obtained performing meta-analysis using the inverse of variance method and the random effects model (except SA1, where fixed effects model was used) on an ITT basis. Sensitivity analysis was conducted using the same statistical methods than the main analysis, but modifying the parameter specified in each of the five performed analyses (SA1 to SA5): Main analysis (all studies included[25–43]). SA1: Use of fixed-effects model instead of random effects (all studies included[25–43]). SA2: Exclusion of studies with crossover design (5 studies, 6 publications, excluded[25,28,29,33,34,43]). SA3: Exclusion of studies with a sample size of 50 patients or fewer (5 studies, 6 publications, excluded[25,28,33,34,41,43]). SA4: Exclusion of studies with a length of treatment of 4 weeks or less (6 studies, 7 publications, excluded[25,28,29,31,33,34,43]). SA5: Exclusion of studies with a high risk of bias (7 studies, 9 publications, excluded[25–27,33,34,37,41–43]). Not estimable: Not available studies in the intervention group. Results with statistical significance shown in bold type.
SA = Sensitivity analysis. RR = rate ratio.

		**RR (95% CI)**					
**Outcome**	**Intervention**	**Main analysis**	**SA1**	**SA2**	**SA3**	**SA4**	**SA5**
Total adverse events	Cannabis extract	1.51 (0.87 to 2.63)	**1.74** (**1.53 to 1.99**)	**2.09** (**1.28 to 3.42**)	1.41 (0.74 to 2.66)	**2.09** (**1.28 to 3.42**)	1.24 (0.26 to 5.93)
	Nabiximols	**1.80** (**1.53 to 2.12**)	**1.85** (**1.68 to 2.03**)	**1.72** (**1.48 to 2.01**)	**1.72** (**1.48 to 2.01**)	**1.74** (**1.47 to 2.06**)	**1.82** (**1.60 to 2.09**)
	Dronabinol	**1.62** (**1.12 to 2.34**)	**1.42** (**1.29 to 1.56**)	**1.42** (**1.07 to 1.88**)	**1.42** (**1.07 to 1.88**)	**1.42** (**1.07 to 1.88**)	**3.43** (**2.16 to 5.46**)
	Cannabinoids	**1.72** (**1.46 to 2.02**)	**1.65** (**1.55 to 1.75**)	**1.70** (**1.47 to 1.98**)	**1.61** (**1.37 to 1.89**)	**1.72** (**1.47 to 2.00**)	**1.80** (**1.45 to 2.24**)
Serious adverse events	Cannabis extract	0.99 (0.26 to 3.74)	0.82 (0.40 to 1.70)	0.99 (0.26 to 3.74)	0.99 (0.26 to 3.74)	0.99 (0.26 to 3.74)	2.19 (0.57 to 8.46)
	Nabiximols	1.43 (0.66 to 3.09)	**1.60** (**1.002 to 2.56**)	1.38 (0.60 to 3.15)	1.38 (0.60 to 3.15)	1.38 (0.60 to 3.15)	1.18 (0.49 to 2.85)
	Dronabinol	1.21 (0.89 to 1.63)	1.21 (0.89 to 1.63)	1.20 (0.88 to 1.62)	1.20 (0.88 to 1.62)	1.20 (0.88 to 1.62)	3.00 (0.12 to 73.64)
	Cannabinoids	1.23 (0.82 to 1.85)	1.25 (0.98 to 1.58)	1.20 (0.78 to 1.84)	1.20 (0.78 to 1.84)	1.20 (0.78 to 1.84)	1.35 (0.67 to 2.71)
Withdrawals due to adverse events	Cannabis extract	**3.11** (**1.54 to 6.28**)	**3.11** (**1.54 to 6.28**)	**3.09** (**1.50 to 6.36**)	**3.11** (**1.54 to 6.28**)	**3.09** (**1.50 to 6.36**)	**3.14** (**1.53 to 6.48**)
	Nabiximols	**2.20** (**1.34 to 3.59**)	**2.20** (**1.34 to 3.59**)	**2.13** (**1.29 to 3.50**)	**2.13** (**1.29 to 3.50**)	**2.11** (**1.27 to 3.49**)	**1.99** (**1.20 to 3.31**)
	Dronabinol	**4.12** (**2.39 to 7.11**)	**4.12** (**2.39 to 7.11**)	**4.12** (**2.39 to 7.11**)	**4.12** (**2.39 to 7.11**)	**4.12** (**2.39 to 7.11**)	Not estimable
	Nabilone	2.63 (0.11 to 64.44)	2.63 (0.11 to 64.44)	2.63 (0.11 to 64.44)	Not estimable	2.63 (0.11 to 64.44)	Not estimable
	Cannabinoids	**2.95** (**2.14 to 4.07**)	**2.95** (**2.14 to 4.07**)	**2.91** (**2.10 to 4.03**)	**2.92** (**2.11 to 4.05**)	**2.91** (**2.10 to 4.04**)	**2.32** (**1.53 to 3.51**)
Dizziness or vertigo	Cannabis extract	2.51 (0.84 to 7.47)	**3.28** (**2.39 to 4.49**)	**5.20** (**2.23 to 12.12**)	2.62 (0.74 to 9.21)	**5.20** (**2.23 to 12.12**)	2.17 (0.15 to 31.20)
	Nabiximols	**3.33** (**2.55 to 4.34**)	**3.33** (**2.55 to 4.34**)	**3.29** (**2.50 to 4.33**)	**3.29** (**2.50 to 4.33**)	**3.29** (**2.47 to 4.37**)	**3.22** (**2.45 to 4.25**)
	Dronabinol	**4.00** (**2.43 to 6.58**)	**4.10** (**3.00 to 5.62**)	**4.15** (**2.97 to 5.80**)	**4.15** (**2.97 to 5.80**)	**4.15** (**2.97 to 5.80**)	**5.20** (**2.00 to 13.54**)
	Cannabinoids	**3.40** (**2.55 to 4.53**)	**3.52** (**2.97 to 4.18**)	**3.83** (**3.20 to 4.59**)	**3.42** (**2.52 to 4.65**)	**3.86** (**3.17 to 4.69**)	**3.22** (**2.06 to 5.04**)
							(continue)

© 2018 Torres-Moreno MC et al. *JAMA Network Open.*

**eTable 4: Sensitivity analysis results for tolerability outcomes** *(continued)*							
		**RR (95% CI)**					
**Outcome**	**Intervention**	**Main analysis**	**SA1**	**SA2**	**SA3**	**SA4**	**SA5**
Dry mouth	Cannabis extract	**3.17 (1.91 to 5.25)**	**3.17 (1.91 to 5.25)**	**3.18 (1.89 to 5.35)**	**3.16 (1.89 to 5.28)**	**3.18 (1.89 to 5.35)**	**3.15 (1.58 to 6.25)**
	Nabiximols	**2.30 (1.42 to 3.73)**	**2.30 (1.42 to 3.73)**	**2.20 (1.34 to 3.59)**	**2.20 (1.34 to 3.59)**	**2.11 (1.28 to 3.48)**	**2.14 (1.28 to 3.60)**
	Dronabinol	**4.32 (2.12 to 8.81)**	**4.32 (2.12 to 8.81)**	**4.12 (1.93 to 8.79)**	**4.12 (1.93 to 8.79)**	**4.12 (1.93 to 8.79)**	7.00 (0.36 to 135.53)
	Cannabinoids	**2.94 (2.15 to 4.03)**	**2.94 (2.15 to 4.03)**	**2.84 (2.06 to 3.92)**	**2.84 (2.06 to 3.91)**	**2.80 (2.02 to 3.88)**	**2.49 (1.67 to 3.73)**
Fatigue	Cannabis extract	**2.60 (1.22 to 5.58)**	**2.60 (1.22 to 5.58)**	**2.60 (1.22 to 5.58)**	**2.60 (1.22 to 5.58)**	**2.60 (1.22 to 5.58)**	**2.60 (1.22 to 5.58)**
	Nabiximols	**1.64 (1.17 to 2.28)**	**1.64 (1.17 to 2.28)**	**1.70 (1.12 to 2.56)**	**1.70 (1.12 to 2.56)**	**1.80 (1.13 to 2.86)**	**1.60 (1.08 to 2.37)**
	Dronabinol	1.09 (0.74 to 1.60)	1.09 (0.74 to 1.60)	1.06 (0.72 to 1.56)	1.06 (0.72 to 1.56)	1.06 (0.72 to 1.56)	5.00 (0.24 to 104.14)
	Cannabinoids	**1.61 (1.18 to 2.21)**	**1.46 (1.15 to 1.85)**	**1.61 (1.14 to 2.30)**	**1.61 (1.14 to 2.30)**	**1.67 (1.14 to 2.43)**	**1.76 (1.25 to 2.47)**
Feeling drunk	Nabiximols	3.70 (0.70 to 19.55)	3.70 (0.70 to 19.55)	3.70 (0.70 to 19.55)	3.70 (0.70 to 19.55)	**8.01 (1.0001 to 64.14)**	3.70 (0.70 to 19.55)
	Dronabinol	11.00 (0.61 to 198.93)	11.00 (0.61 to 198.93)	Not estimable	Not estimable	Not estimable	11.00 (0.61 to 198.93)
	Cannabinoids	**4.85 (1.15 to 20.53)**	**4.85 (1.15 to 20.53)**	3.70 (0.70 to 19.55)	3.70 (0.70 to 19.55)	**8.01 (1.0001 to 64.14)**	**4.85 (1.15 to 20.53)**
Impaired balance or ataxia	Cannabis extract	3.50 (0.18 to 67.77)	3.50 (0.18 to 67.77)	Not estimable	Not estimable	Not estimable	Not estimable
	Nabiximols	**2.93 (1.04 to 8.27)**	**2.93 (1.04 to 8.27)**	**2.93 (1.04 to 8.27)**	**2.93 (1.04 to 8.27)**	**2.93 (1.04 to 8.27)**	**3.81 (1.27 to 11.42)**
	Dronabinol	1.28 (0.90 to 1.81)	1.28 (0.90 to 1.81)	1.25 (0.88 to 1.78)	1.25 (0.88 to 1.78)	1.25 (0.88 to 1.78)	5.00 (0.24 to 104.14)
	Cannabinoids	**1.40 (1.01 to 1.95)**	**1.40 (1.01 to 1.95)**	1.37 (0.98 to 1.91)	1.37 (0.98 to 1.91)	1.37 (0.98 to 1.91)	3.93 (1.40 to 11.04)
Memory impairment	Nabiximols	**4.93 (1.07 to 22.70)**	**4.93 (1.07 to 22.70)**	**4.93 (1.07 to 22.70)**	**4.93 (1.07 to 22.70)**	**4.93 (1.07 to 22.70)**	**4.93 (1.07 to 22.70)**
	Cannabinoids	**4.93 (1.07 to 22.70)**	**4.93 (1.07 to 22.70)**	**4.93 (1.07 to 22.70)**	**4.93 (1.07 to 22.70)**	**4.93 (1.07 to 22.70)**	**4.93 (1.07 to 22.70)**
							(continue)

© 2018 Torres-Moreno MC et al. *JAMA Network Open.*

**eTable 4: Sensitivity analysis results for tolerability outcomes** *(continued)*							
		**RR (95% CI)**					
**Outcome**	**Intervention**	**Main analysis**	**SA1**	**SA2**	**SA3**	**SA4**	**SA5**
Somnolence	Cannabis extract	1.32 (0.95 to 1.83)	1.32 (0.95 to 1.83)	1.32 (0.94 to 1.85)	1.32 (0.94 to 1.85)	1.32 (0.94 to 1.85)	1.50 (0.06 to 36.82)
	Nabiximols	**3.47** (**2.10 to 5.73**)	**3.47** (**2.10 to 5.73**)	**3.50** (**2.04 to 6.00**)	**3.50** (**2.04 to 6.00**)	**3.42** (**1.98 to 5.93**)	**3.48** (**1.99 to 6.07**)
	Dronabinol	0.55 (0.06 to 4.74)	1.08 (0.77 to 1.53)	1.12 (0.79 to 1.58)	1.12 (0.79 to 1.58)	1.12 (0.79 to 1.58)	Not estimable
	Cannabinoids	**1.87** (**1.24 to 2.81**)	**1.46** (**1.18 to 1.81**)	**1.92** (**1.25 to 2.96**)	**1.88** (**1.25 to 2.82**)	**1.88** (**1.22 to 2.90**)	**3.39** (**1.96 to 5.88**)

© 2018 Torres-Moreno MC et al. *JAMA Network Open.*

3. Supplementary eFigures

eFigure 1: Risk of bias summary of the included studies

Review authors' judgements about each risk of bias item for each included study.

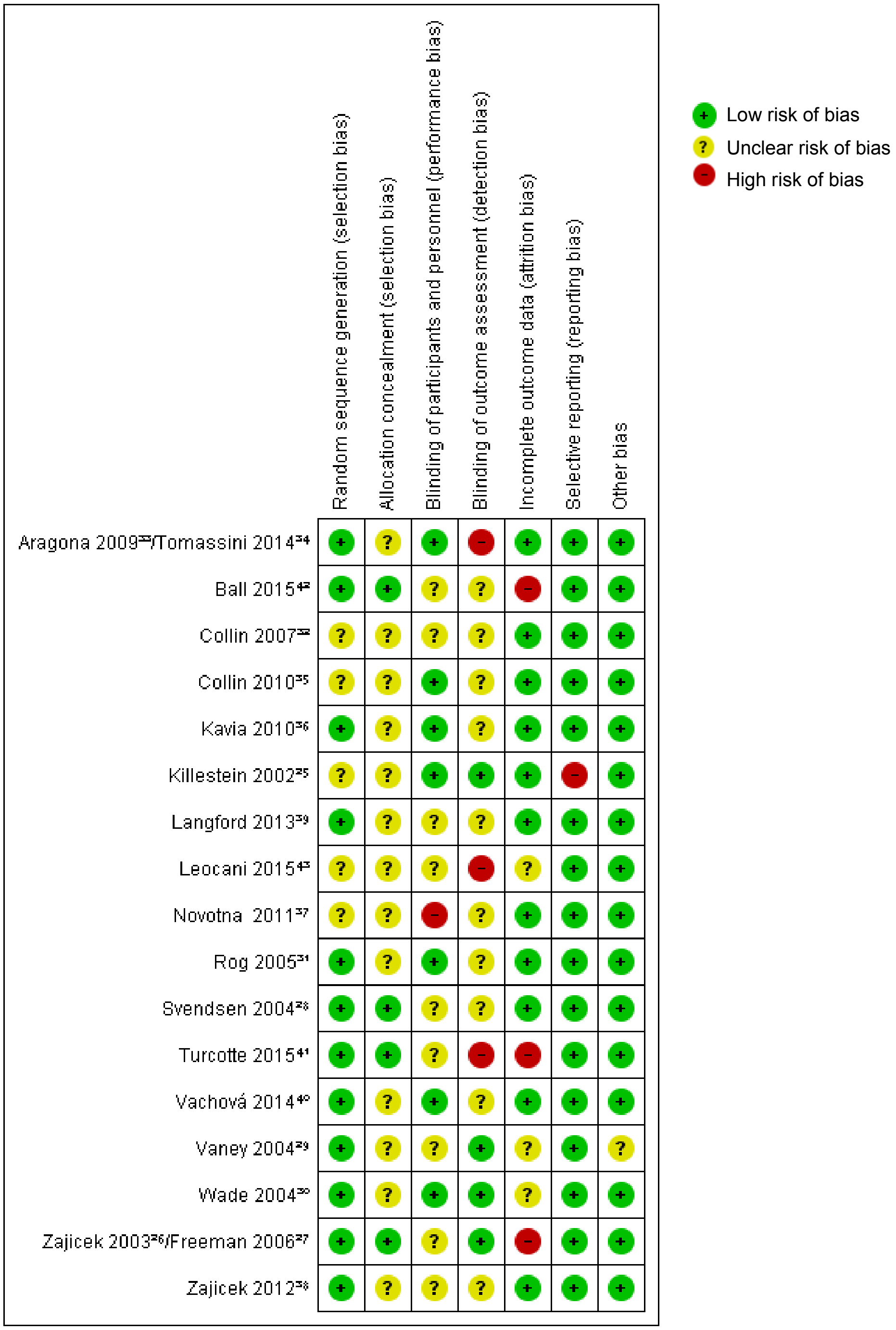

© 2018 Torres-Moreno MC et al. *JAMA Network Open.*

eFigure 2: Risk of bias graph of the included studies

Review authors' judgements about each risk of bias item presented as percentages across all included studies.[25-43]

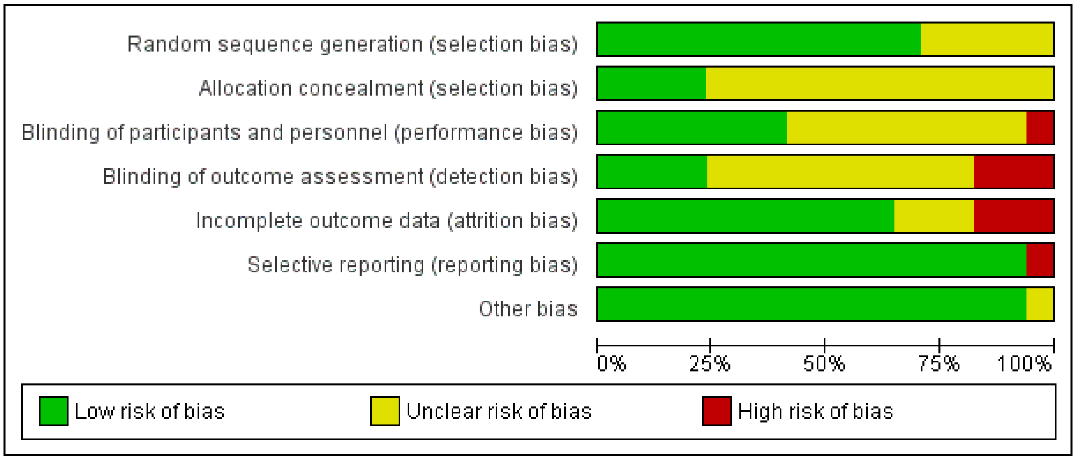

© 2018 Torres-Moreno MC et al. *JAMA Network Open.*

eFigure 3: Funnel plots for efficacy outcomes

Panels 3a) Spasticity (Ashworth), 3b) Spasticity (subjective), 3c) Pain, and 3d) Bladder dysfunction

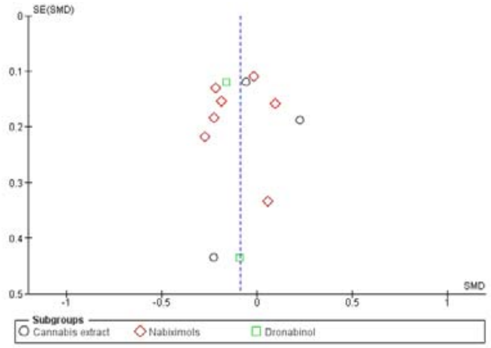

Panel 3a) Spasticity (Ashworth)

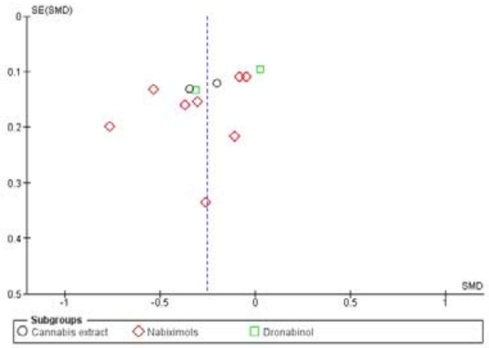

Panel 3b) Spasticity (subjective)

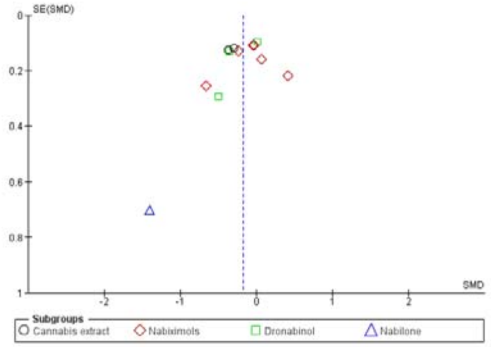

Panel 3c) Pain

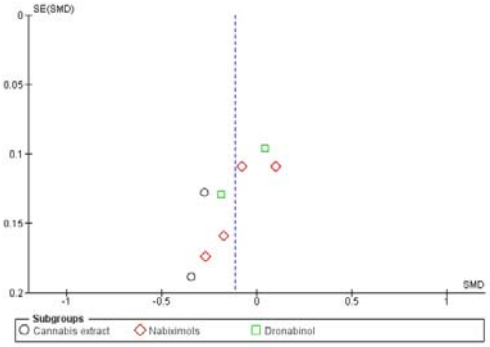

Panel 3d) Bladder dysfunction

© 2018 Torres-Moreno MC et al. *JAMA Network Open.*

eFigure 4: Funnel plots for tolerability outcomes

Panels 4a) Total adverse events, 4b) Serious adverse events, 4c) Withdrawals due to adverse events, 4d) Dizziness/vertigo, 4e) Dry mouth, 4f) Fatigue, 4g) Feeling drunk, 4h) Impaired balance/ataxia, 4i) Memory impairment, and 4j) Somnolence

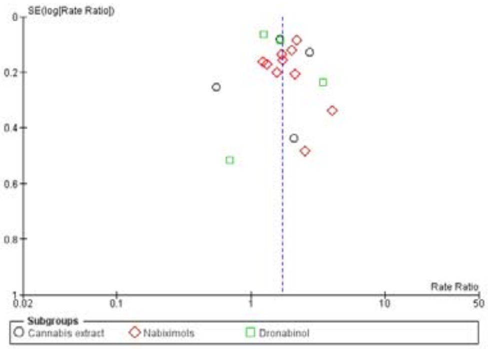

Panel 4a) Total adverse events

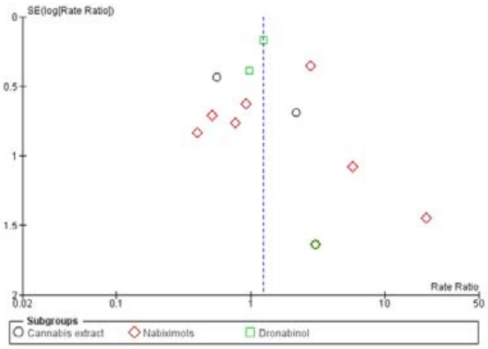

Panel 4b) Serious adverse events

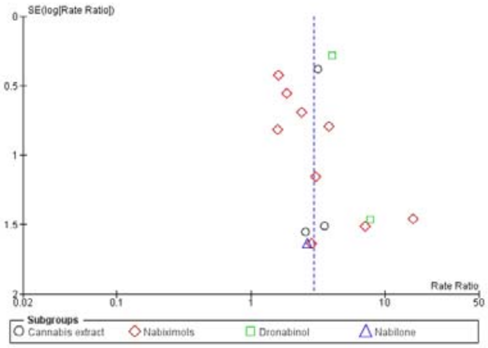

Panel 4c) Withdrawals due to adverse events

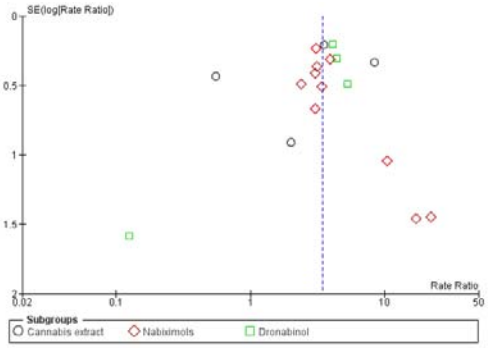

Panel 4d) Dizziness or vertigo

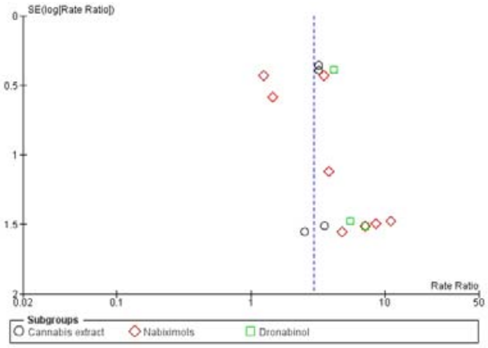

Panel 4e) Dry mouth

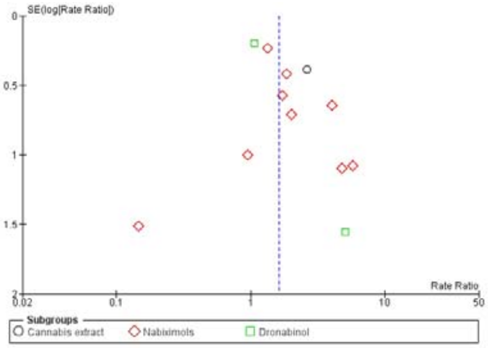

Panel 4f) Fatigue

(continue)

© 2018 Torres-Moreno MC et al. *JAMA Network Open.*

eFigure 4: Funnel plots for tolerability outcomes *(continued)*

Panels 4a) Total adverse events, 4b) Serious adverse events, 4c) Withdrawals due to adverse events, 4d) Dizziness/vertigo, 4e) Dry mouth, 4f) Fatigue, 4g) Feeling drunk, 4h) Impaired balance/ataxia, 4i) Memory impairment, and 4j) Somnolence

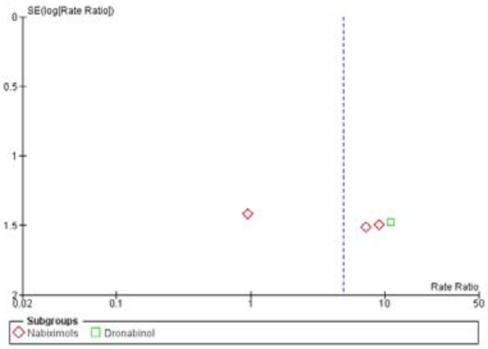

Panel 4g) Feeling drunk

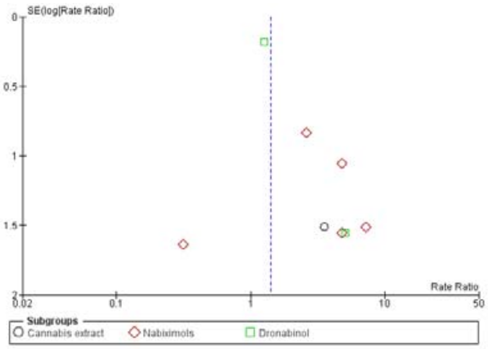

Panel 4h) Impaired balance or ataxia

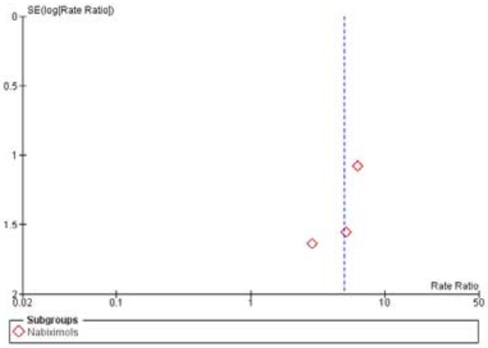

Panel 4i) Memory impairment

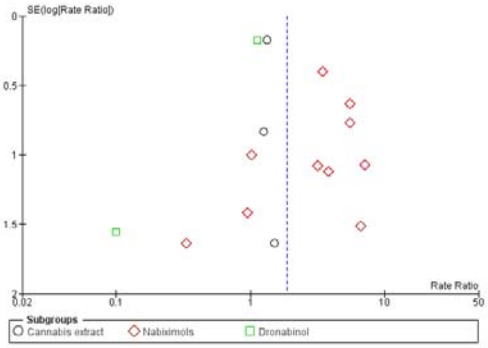

Panel 4j) Somnolence

© 2018 Torres-Moreno MC et al. *JAMA Network Open.*
